# Colonization of root endophytic fungus *Serendipita indica* improves drought tolerance of *Pinus taeda* seedlings by regulating metabolome and proteome

**DOI:** 10.3389/fmicb.2024.1294833

**Published:** 2024-03-15

**Authors:** Chu Wu, Yujie Yang, Yun Wang, Wenying Zhang, Honggang Sun

**Affiliations:** ^1^College of Horticulture and Gardening, Yangtze University, Jingzhou, Hubei, China; ^2^College of Life Sciences, Yangtze University, Jingzhou, Hubei, China; ^3^College of Agricultural Sciences, Yangtze University, Jingzhou, Hubei, China; ^4^Research Institute of Subtropical Forestry, Chinese Academy of Forestry, Hangzhou, China

**Keywords:** forest trees, drought stress, metabolome, proteome, beneficial microorganisms

## Abstract

*Pinus taeda* is an important forest tree species for plantations because of its rapid growth and high yield of oleoresins. Although *P. taeda* plantations distribute in warm and wet southern China, drought, sometime serious and long time, often occurs in the region. To explore drought tolerance of *P. taeda* and usage of beneficial microorganisms, *P. taeda* seedlings were planted in pots and were inoculated with root endophytic fungus *Serendipita indica* and finally were treated with drought stress for 53 d. Metabolome and proteome of their needles were analyzed. The results showed that *S. indica* inoculation of *P. taeda* seedlings under drought stress caused great changes in levels of some metabolites in their needles, especially some flavonoids and organic acids. Among them, the levels of eriocitrin, *trans*-aconitic acid, vitamin C, uric acid, alpha-ketoglutaric acid, vitamin A, stachydrine, coumalic acid, itaconic acid, calceolarioside B, 2-oxoglutaric acid, and citric acid were upregulated more than three times in inoculated seedlings under drought stress, compared to those of non-inoculated seedlings under drought stress. KEGG analysis showed that some pathways were enriched in inoculated seedlings under drought stress, such as flavonoid biosynthesis, ascorbate and aldarate metabolism, C5-branched dibasic acid metabolism. Proteome analysis revealed some specific differential proteins. Two proteins, namely, H9X056 and H9VDW5, only appeared in the needles of inoculated seedlings under drought stress. The protein H9VNE7 was upregulated more than 11.0 times as that of non-inoculated seedlings under drought stress. In addition, *S. indica* inoculation increased enrichment of water deficient-inducible proteins (such as LP3-1, LP3-2, LP3-3, and dehydrins) and those involved in ribosomal structures (such as A0A385JF23). Meanwhile, under drought stress, the inoculation caused great changes in biosynthesis and metabolism pathways, mainly including phenylpropanoid biosynthesis, cutin, suberine and wax biosynthesis, and 2-oxocarboxylic acid metabolism. In addition, there were positive relationships between accumulation of some metabolites and enrichment of proteins in *P. taeda* under drought stress. Altogether, our results showed great changes in metabolome and proteome in inoculated seedlings under drought stress and provided a guideline to further study functions of metabolites and proteins, especially those related to drought stress.

## 1 Introduction

Loblolly pine (*Pinus taeda*) is native to the southeastern United States (US) and is an important forest tree species in the southern mixed pine-oak forest (Kricher and Morrison, 1998) and the Coastal Plain oak-pine forests (Keyser et al., 2016) in the southern and the southeastern US, respectively. The forest species is the most-planted forest tree species in North America and managed for wood production (Matallana-Ramirez et al., 2021; McKeand et al., 2021). There are approximately 25 million hectares of planted trees in US in 2011, of which loblolly pine represents 10 million hectares, making it the most-planted tree species in the country (Gonzalez-Benecke et al., 2017). Planted forests account for 22% of all forested area in the southeastern US (Gonzalez-Benecke et al., 2017), and they play a large role in meeting the nation’s wood and fiber demand. Gonzalez-Benecke et al. (2017) predicted that an increase in productivity can be expected for a large majority of the planted loblolly pine stands in the southeastern US during 21th century. Because of its rapid growth and high oleoresins yield, loblolly pine was also introduced to other countries around the world. Terpenoid oleoresins can be converted into rosin and turpentine, which show wide and strong commercial uses such as adhesives, inks, emulsifiers, solvents, fragrances, and resins (da Silva Rodrigues-Corrêa et al., 2012). According to an estate in 2004, approximately 30,000 tons of pinenes were consumed only by the flavor and fragrance industry per year to produce a range of products (Swift, 2004); thus, such great requirement improves oleoresins production from *Pinus* species around the world. Since loblolly pine was introduced into China in thirties of last century, the forest tree species became more and more important for plantations along with slash pine (*Pinus elliottii*) in southern China, especially the valleys of the Yangtze River and Zhujiang River. Approximately 0.6 million of tons of resin was harvested each year in China, accounting for 60% of the global gum resin yield and about half of the worldwide turpentine trade (McConnell et al., 2021; Yi et al., 2021), most of which was harvested from loblolly pine and slash pine.

Along with other environmental factors, such as soil chemistry, annual average temperature, and incident radiation, water availability is one of the principal physical factors limiting primary productivity of terrestrial plants. Water availability was identified as the most influential single variable for estimating the net primary productivity of terrestrial ecosystems (Churkina et al., 1999). Thus, drought stress affects the net primary productivity of terrestrial ecosystems, especially forest ecosystems because of their important ecosystem system service functions. For *P. taeda* in a 35-year-old stand, oleoresin yield was closely associated with periods of calculated moderate soil water deficit and presumed growth (Lorio and Sommers, 1986). In such stand, moderate seasonal water deficits limited pine growth but did not limit photosynthesis and translocation of photosynthates and favor differentiation processes, such as oleoresin synthesis (Lorio and Sommers, 1986). However, serious and long-term drought certainly reduces both pine growth and oleoresin yield. In fact, drought stress is the principal cause of seedling mortality in pine forests of the southeastern US (Lorenz et al., 2006). Although annual precipitation is enough for growth and development of loblolly pine in southern and southwestern China, climate change often caused seasonal drought in some areas in this region. For example, persistent drought in 2006 summer caused pine death in large scale in Sichuan province, localized in southwestern China (Wang, 2012). From the autumn to the spring in 2009/2010 and 2011/2012, severe drought occurred in southwestern China, especially in Yunnan province (Sun et al., 2014). Such severe drought caused death of seedlings and saplings of *Pinus kesiya* var. *langbianensis* and *Pinus yunnanensis* in this province. Subsequently, severe drought occurred in Fujian province, located in southeastern China, in March–June of 2018 (He F. et al., 2022). In the summer of 2022, great reduction in precipitation occurred in the valley of the Yangtze River, which is the longest river in China with an average stream flow of 33,980 m^3^·s^−1^, resulting in flux break of few tributary rivers and partial occurrence of riverbed of the Yangtze River. According to data collected from 1952 to 2010, Um et al. (2022) confirmed that the propagation phenomenon of meteorological to hydrological and agricultural droughts occurred in the valley of the Yangtze River. The concurrent occurrence of drought and pine diseases accelerates pine death. For example, persistent summer drought triggered energy metabolism imbalance and entire wilt and even death of pine trees caused by *Bursaphelenchus xylophilus* in China (Wang, 2012). Therefore, under the background of climate change, forest ecosystems in the valley of the Yangtze River are under threat from severe droughts (Yin et al., 2021).

Plants use different mechanisms to survive under drought stress, including the five aspects: (1) soil water deficit avoidance (e.g., root exploration, water conservation, and phenology); (2) stress avoidance (e.g., osmotic adjustment and root-soil isolation); (3) damage avoidance (i.e., stress tolerance, e.g., leaf orientation, evaporative cooling, and root-to-shoot ratio); (4) damage tolerance (e.g., night-time recovery, heat shock proteins, and dehydrins); (5) unadapted (e.g., death, organ loss, and permanent damage) (Gilbert and Medina, 2016). During water deficient, plants synergistically use these mechanisms to survive, especially under severe drought stress. Facing drought stress, loblolly pine forest farm managers and tree physiologists have to find suitable ways to maintain survival, growth, and oleoresin yield of loblolly pine. The suitable ways include few aspects: (1) to clearly explore the genetic characteristics of loblolly pine (Lorenz et al., 2011; Wegrzyn et al., 2014; Perera et al., 2018; Caballero et al., 2021) and functions of genes and metabolites related to drought stress (González-Martínez et al., 2006; Lorenz et al., 2006; Talbot et al., 2017; Wu et al., 2023), further providing the base for genetic modification of loblolly pine; (2) based on genetic characteristics of loblolly pine, to breed new cultivars of loblolly pine with stronger drought tolerance (Zapata-Valenzuela et al., 2013; Matallana-Ramirez et al., 2021); (3) to take advantage of benefits from beneficial microbes, especially ectomycorrhizal fungi and root endophytic fungi under drought stress (Piculell et al., 2019; Wu et al., 2019; Frank and Garcia, 2021).

Symbiosis between plants and microorganisms occurs wide in natural ecosystems, affects plant terrestrializations (Puginier et al., 2022), evolution (Batstone, 2022; van Galen et al., 2023), and tolerance to abiotic and biotic stress (Zeng et al., 2022), and widens the habitability ranges of plants (Muñoz and Carneiro, 2022). Among all the beneficial microorganisms symbiosing with plants, five families of microorganisms have been greatly paid on attentions, i.e., arbuscular mycorrhizal fungi (Kaur et al., 2022; Razak and Gange, 2023; Chen W. et al., 2023), ectomycorrhizal fungi (Karlsen-Ayala et al., 2022; Jörgensen et al., 2023; Xiao et al., 2023), root endophytic fungi (Manzur et al., 2022; Sun et al., 2022; Qin et al., 2023), dark septate fungi (Gaber et al., 2023; Wang et al., 2023; Chen S. et al., 2023), and plant growth-promoting rhizobacteria (PGPR) (Ahmad et al., 2022; Gowtham et al., 2022; Zhao et al., 2023). These beneficial microorganisms show strong effects on their plant hosts under drought stress (Ahmad et al., 2022; Gowtham et al., 2022; Zhao et al., 2023). In review of such functions, they are often used as components of biofilmed biofertilizers (Das et al., 2017; Kumar et al., 2021; Zahra et al., 2023). Biofilmed biofertilizers have emerged as a new improved inoculant technology to improve efficient nutrition uptake, strengthen management of pests and pathogenic microorganisms, and sustain soil fertility (Das et al., 2017). However, because of their life traits, i.e., obligate biotroph (Zuccaro et al., 2014), it is difficult to proliferate arbuscular mycorrhizal fungi on a large scale in short time. Ectomycorrhizal fungi, root endophytic fungi, and PGPR show their advantage in agricultural application because of their facultative biotroph. The root endophytic fungus *Serendipita indica* (i.e., formerly named as *Piriformospora indica*) can colonize in a wide range of plant hosts, such as *Arabidopsis thaliana* (Opitz et al., 2021), *Juglans regia* (Liu et al., 2021), *Oryza sativa* (Ghorbani et al., 2021), *Platycladus orientalis* (Wu et al., 2019), and tomato (*Solanum lycopersicum*) (De Rocchis et al., 2022), and shows strong effects on their plant hosts, especially improving plant nutrition uptake (Wu et al., 2019) and tolerance to drought stress (Liu et al., 2021; Boorboori and Zhang, 2022). However, it is unclear how *S. indica* regulates responses of plant hosts to drought stress in levels of metabolome and proteome, especially forest tree species. The related mechanisms are not still clear, such as the functions of effector proteins secreted by the fungus. Analysis of metabolome and proteome provides outline of changes in metabolites and proteins in plants under drought stress, and the related results also provide a guide for future research, especially the functions of small metabolites and unique proteins that accumulate to high levels in plants under drought stress.

As mentioned above, beneficial microorganisms show strong ability to improve plant tolerance to drought stress; however, related studies involved in loblolly pine are few, especially those involved in drought tolerance mechanisms. The root endophytic fungus *S. indica* can be used to improve plant tolerance to drought stress, as shown above. In the present study, *S. indica* was used to inoculate with loblolly pine seedlings, and untargeted metabolome and proteome of their needles were analyzed. Our aims are (1) to know changes in metabolome and proteome caused by inoculation of *S. indica* under drought stress and (2) to explore the related mechanisms that are involved in increased drought tolerance caused by *S. indica*.

## 2 Experimental materials and methods

### 2.1 Experimental materials

Seeds of loblolly pine (*Pinus taeda*) came from the Forest Farm of Maple Mountain in Jingdezhen, Jiangxi province, China. These seeds were sterilized and then sown in sterilized sand in big plastic pots. After these seeds were germinated, the pots were transferred to a growth chamber (light period: 16-h light/8-h dark; PPFD: 350 μmol·m^−2^·s^−1^; RH: 80%; temperature: 25°C). When pine seedlings grew with four true needles, they were transplanted into plastic pots (20 cm in diameter and 30 cm in height) containing sterilized cultivation substance (peat: vermiculite = 50: 50, pH ~ 7.0), three pine seedlings per pot. These pots were then transferred into a greenhouse with natural sunlight and controlled temperature (25°C) and relative humidity (~75%). Water was provided according to the moisture of the cultivation substance in the pots. After a month, half of these pots were inoculated with the root endophytic fungus *S. indica*.

*Serendipita indica* strain DSM 11827 was provided by Prof. Ralf Oelmüller (Faculty of Biological Science, Friedrich-Schiller-University Jena, Jena, Germany). The fungus was cultured on potato dextrose agar (PDA) in liquid culture at 180–200 rpm for 7 d (28°C). The hyphae were filtered, rinsed with sterile water three times, and then treated with filter paper. In total, 10 g of fresh hyphae was weighed and made into homogenate. The homogenate was then made to a suspension solution of 1 L, and the suspension solution was used for inoculation of loblolly pine seedlings.

### 2.2 Inoculation and drought stress treatment

When loblolly pine seedlings were inoculated with the suspension solution mentioned above, every pot was injected with 10 mL of the suspension solution at the center of a pot. After inoculation, all the pots with loblolly pine seedlings were cultivated in a glasshouse as mentioned above. After culture of 120 d, drought stress treatment was carried out: 30 pots with inoculated seedlings were divided into two groups, one was treated with drought stress, and another group was watered well; 30 pots with non-inoculated seedlings were also divided into two groups, one group treated with drought stress, and another group watered well. Therefore, four treatments were established, i.e., non-inoculated seedlings under well-watered condition (NI_W treatment), inoculated seedlings under well-watered condition (I_W treatment), non-inoculated seedlings under drought stress (NI_D treatment), and inoculated seedlings under drought stress (I_D treatment). These pots treated with drought stress were provided with 100 mL water once every week. According to the previous trial experiment, 100 mL of water for a pot could maintain approximately 53% field capacity. Well-watered seedlings were provided with enough water once every week at the same time. After drought stress treatment of 53 d, six pots were random chosen from every treatment, and a seedling was random chosen from a pot. Their needles of the six seedlings were harvested and then were rinsed with sterile water three times. The needles from the two random chosen seedlings were combined into a sample. Thus, three samples were harvested for each treatment, and total 12 samples harvested for all the four treatments. These samples were immediately treated with liquid nitrogen and stored under −80°C for further analysis.

### 2.3 Examination of *Serendipita indica* infection

Twenty days after inoculation of *S. indica* with loblolly pine seedlings*, S. indica* infection was examined according to the method introduced by Xu et al. (2023).

### 2.4 Methods of untargeted metabolomic analysis

Needle tissues (100 mg) were individually grounded with liquid nitrogen, and the homogenate was resuspended with prechilled 80% methanol by well vortex. The samples were incubated on ice for 5 min and then were centrifuged at 15,000 *g* for 20 min (4°C). Some of supernatant was diluted to final concentration containing 53% methanol by LC–MS grade water. The samples were subsequently transferred to fresh Eppendorf tubes and then were centrifuged at 15000 g for 20 min (4°C). Finally, the supernatant was injected into the LC–MS/MS system analysis (Want et al., 2013). Subsequently, UHPLC–MS/MS analysis, data processing and metabolite identification, and data analysis were carried out according to the previous introduction (Wu et al., 2023).

### 2.5 Methods of untargeted proteome analysis

#### 2.5.1 Extraction of total proteins in leaves of *Pinus taeda* seedlings

The sample was ground individually in liquid nitrogen and lysed with SDT lysis buffer (containing 100 mM NaCl) and 1/100 volume of DTT, followed by 5 min of ultrasonication on ice. After reacting at 95°C for 8–15 min and ice bath for 2 min, the lysate was centrifuged at 12,000 *g* for 15 min at 4°C. The supernatant was alkylated with sufficient IAM for 1 h at room temperature in the dark. Then, the samples were completely mixed with four times the volume of pre-cooled acetone by vortexing and incubated at-20°C for at least 2 h. The samples were then centrifuged at 12,000 *g* for 15 min at 4°C, and the precipitation was collected. After washing with 1 mL cold acetone, the pellet was dissolved by dissolution buffer (DB buffer).

#### 2.5.2 Protein quality test

BSA standard protein solution was prepared according to the instructions of Bradford protein quantitative kit, with gradient concentration ranging from 0 to 0.5 g·L^−1^. BSA standard protein solutions and sample solutions with different dilution multiples were added into 96-well plate to fill up the volume to 20 μL, respectively. Each gradient was repeated three times. The plate was added 180 μL G250 dye solution quickly and placed at room temperature for 5 min, and the absorbance at 595 nm was detected. The standard curve was drawn with the absorbance of standard protein solution, and the protein concentration of the sample was calculated. 20 μg of the protein sample was loaded to 12% SDS-PAGE gel electrophoresis, wherein the concentrated gel was performed at 80 V for 20 min, and the separation gel was performed at 120 V for 90 min. The gel was stained by Coomassie brilliant blue R-250 and decolored until the bands were visualized clearly.

#### 2.5.3 Trypsin treatment

Trypsin treatment was carried out according to the method introduced by Zhang et al. (2016). Each protein sample was taken, and the volume was made up to 100 μL with DB lysis buffer (8 M Urea, 100 mM TEAB, pH 8.5), trypsin and 100 mM TEAB buffer were added, and the sample was mixed and digested at 37°C for 4 h. Then, trypsin and CaCl_2_ were added digested overnight. Formic acid was mixed with digested sample, adjusted pH under 3, and centrifuged at 12,000 *g* for 5 min at room temperature. The supernatant was slowly loaded to the C18 desalting column, washed with washing buffer (0.1% formic acid, 3% acetonitrile) three times, and then added elution buffer (0.1% formic acid, 70% acetonitrile). The eluents of each sample were collected and lyophilized (Zhang et al., 2016).

#### 2.5.4 Separation of fractions (high-depth quantification)

Mobile phases A (2% acetonitrile, adjusted pH to 10.0 using ammonium hydroxide) and B (98% acetonitrile, adjusted pH to 10.0 using ammonium hydroxide) were used to develop a gradient elution. The lyophilized powder was dissolved in solution A and centrifuged at 12,000 *g* for 10 min at room temperature. The sample was fractionated using a C18 column (Waters BEH C18, 4.6 × 250 mm, 5 μm) on a Rigol L3000 HPLC system, and the column oven was set as 45°C. The detail of elution gradient is shown in Supplementary Table S1. The eluates were monitored at UV 214nm, collected in a tube per minute, and combined into 10 fractions finally. All fractions were dried under vacuum and then reconstituted in 0.1% (v/v) formic acid (FA) in water.

#### 2.5.5 LC–MS/MS analysis

UHPLC–MS/MS analyses were performed using an EASY-nLC™ 1,200 UHPLC-HFX system (Thermo Fisher, Germany) coupled with a Q Exactive™ HF-X mass spectrometer (Thermo Fisher, Germany) in Novogene Co., Ltd. (Beijing, China). First, mobile phase A (100% water, 0.1% formic acid) and B (80% acetonitrile, 0.1% formic acid) solutions were prepared. The lyophilized powder was dissolved in 10 μL of solution A and centrifuged at 14,000 *g* for 20 min at 4°C, and 1 μg of the supernatant was injected into a home-made C18 Nano-Trap column (4.5 cm × 75 μm, 3 μm). The temperature of the column oven was set to 55°C. Peptides were separated in a home-made analytical column (15 cm × 150 μm, 1.9 μm), using a linear gradient elution as listed in Supplementary Table S1. The separated peptides were analyzed by Q Exactive™ HF-X mass spectrometer, with ion source of Nanospray Flex™(ESI), spray voltage of 2.1 kV, and ion transport capillary temperature of 320°C. Full scan ranges from *m/z* 350 to 1,500 with resolution of 60,000 (at *m/z* 200), an automatic gain control (AGC) target value was 3 × 10^6^, and a maximum ion injection time was 20 ms. The top 40 precursors of the highest abundant in the full scan were selected and fragmented by higher energy collisional dissociation (HCD) and analyzed in MS/MS, where resolution was 15,000 (at *m/z* 200), the automatic gain control (AGC) target value was 1 × 10^5^, the maximum ion injection time was 45 ms, a normalized collision energy was set as 27%, an intensity threshold was 2.2 × 10^4^, and the dynamic exclusion parameter was 20 s. The raw data of MS detection were named as “.raw.”

#### 2.5.6 Data analysis

At first, the identification and quantitation of protein were carried out. The resulting spectra were searched against *** database by the search engines: Proteome Discoverer (Thermo, HFX and 480) or MaxQuant (Bruker, Tims). The search parameters of Proteome Discoverer were set as follows: Mass tolerance for precursor ion was 10 ppm, and mass tolerance for product ion was 0.02 Da. Carbamidomethyl was specified as fixed modifications, and oxidation of methionine (M) was specified as dynamic modification and loss of methionine at the N-Terminal. A maximum of two missed cleavage sites were allowed. The search parameters of MaxQuant were set as follows: Mass tolerance for precursor ion was 20 ppm, and mass tolerance for product ion was 0.05 Da. Carbamidomethyl was specified as fixed modifications, oxidation of methionine (M) was specified as dynamic modification, and acetylation was specified as N-terminal modification. A maximum of two missed cleavage sites were allowed. To improve the quality of analysis results, the software PD or MaxQuant further filtered the retrieval results: Peptide spectrum matches (PSMs) with a credibility of more than 99% were identified PSMs. The identified protein contained at least one unique peptide. The identified PSMs and protein were retained and performed with FDR no more than 1.0%. The protein quantitation results were statistically analyzed by *t*-test. The proteins whose quantitation was significantly different between experimental and control groups (*p* < 0.05) and |log2FC| > * [FC > * or FC < * (fold change, FC)] were defined as differentially expressed proteins (DEPs).

Next, the functional analysis of proteins and DEPs was carried out. Gene Ontology (GO) and InterPro (IPR) functional analysis were conducted using the InterProScan program against the non-redundant protein database (including Pfam, PRINTS, ProDom, SMART, ProSite, PANTHER) (Jones et al., 2014), and the databases of Clusters of Orthologous Groups (COG) and Kyoto Encyclopedia of Genes and Genomes (KEGG) were used to analyze the protein families and pathways. DEPs were used for volcanic map analysis, cluster heat map analysis, and enrichment analysis of GO, IPR, and KEGG (Huang et al., 2009). The probable protein–protein interactions were predicted using the STRING-db server (Franceschini et al., 2013)^1^.

### 2.6 Correlation analysis on metabolites–proteins

Correlation analysis was carried out based on Pearson’s coefficients of correlation to measure the correlation extents among top 50 differential proteins and top 50 differential metabolites with significant differences in the four treatments. When Pearson’s coefficients of correlation were less than 0, the correlation was negative, while when Pearson’s coefficients of correlation were more than 0, the correlation was positive. Network figures showed the visualized relationships between these proteins and metabolites with significant differences. Top 10 differential proteins and top 5 differential metabolites were selected for visualized network figures. Differential metabolites were marked as yellow, and differential proteins were marked as blue. Lines showed their respective relationships: Red and blue lines showed positive and negative relationships between these differential metabolites and proteins, respectively.

## 3 Results

### 3.1 Colonization of *Serendipita indica* in roots of *Pinus taeda* seedlings

**Figure 1:**
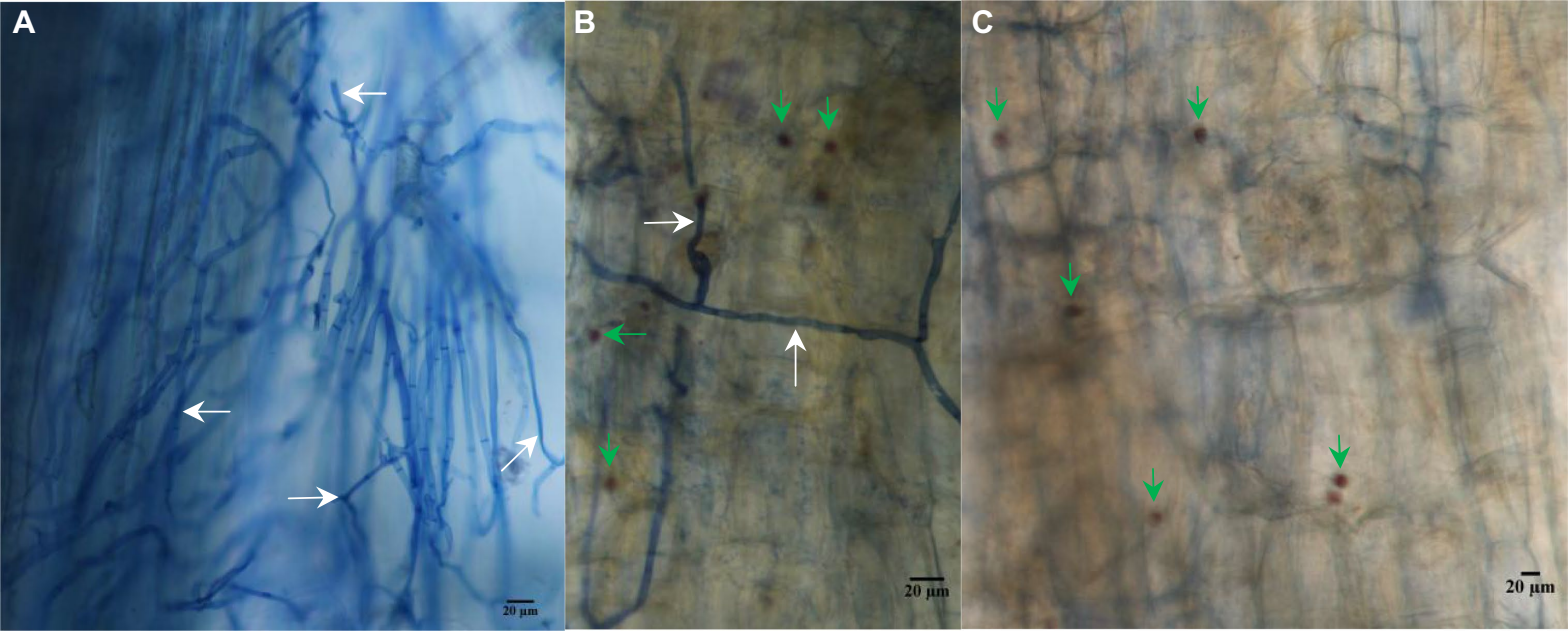
Infection of *Serendipita indica* in roots of *Pinus taeda* plants. **(A)** *S. serendipita* hyphae (shown by white arrows) on the roots of *Pinus taeda* plants. **(B)** Hyphae (shown by white arrows) and spores (shown by green arrows) of *Serendipita indica* on the root surface of *Pinus taeda* plants. **(C)** Spores (shown by green arrows) of *Serendipita indica* in the root cortex of *Pinus taeda* plants. Bar = 20 μm.

Just like its ability to colonize in roots of some plant species (Wu et al., 2019; Liu et al., 2021; De Rocchis et al., 2022), *S. indica* colonized in the roots of *P. taeda* seedlings (Figure 1). Its hyphae and spores were found on the root surface and cortex of *P. taeda* seedlings, as shown in Figures 1A,B, respectively.

### 3.2 Effects of *Serendipita indica* colonization on untargeted metabolome

#### 3.2.1 Outline of differential metabolites under the four treatments

**Figure 2:**
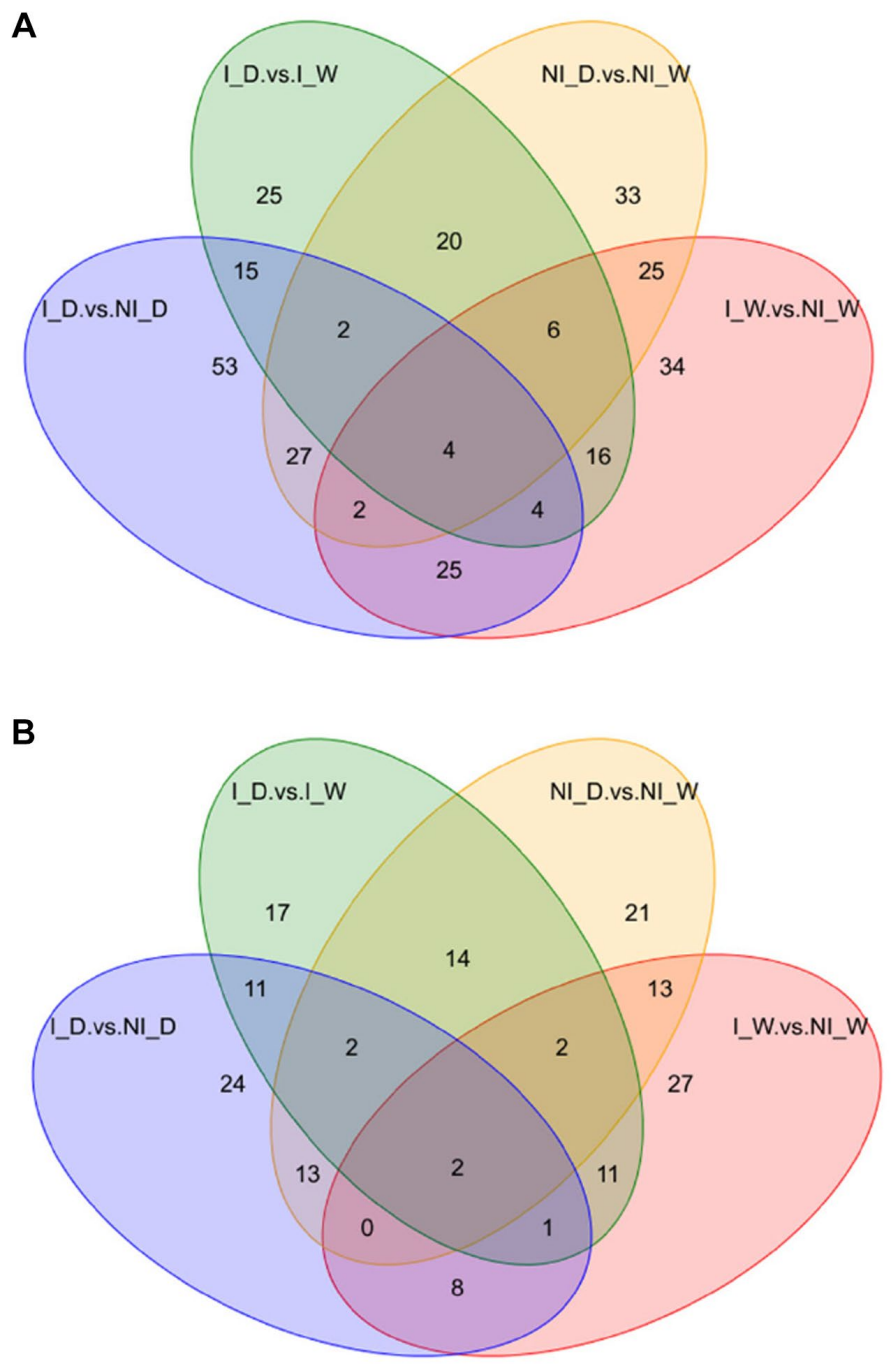
Venn diagram of differential metabolites under positive **(A)** and negative **(B)** ionization modes. I_W: inoculated seedlings under well-watered condition; NI_W: non-inoculated seedlings under well-watered condition; I_D: inoculated seedlings under drought stress; I_W: inoculated seedlings under well-watered condition; NI_D: non-inoculated seedlings under drought stress.

After examination of quality control and identification of differential metabolites (Supplementary material 1, including Supplementary Tables S2, S3), Venn maps were used to show that the four comparisons possessed different numbers of unique differential metabolites. Under positive ionization mode, 34, 33, 25, and 53 unique differential metabolites occurred in the four comparisons, i.e., I_W vs. NI_W, NI_D vs. NI_W, I_D vs. I_W, and I_D vs. NI_D, respectively (Figure 2A; Supplementary Table S4). Under negative ionization mode, 27, 21, 17, and 24 unique differential metabolites occurred in the four comparisons, respectively (Figure 2B; Supplementary Table S4). Combining the numbers of these unique differential metabolites under positive and negative ionization mode, it was found that the comparison, i.e., I_D vs. NI_D, possessed the greatest number of unique differential metabolites (77 metabolites), suggesting that inoculation of *S. indica* resulted in great changes in metabolites in *P. taeda* seedlings under drought stress. The two metabolites, i.e., gomisin D and vinorelbine tartrate, occurred in all the four comparisons under negative ionization mode, and the other four metabolites, i.e., arachidonic acid methyl ester, LPC 12:0, estriol, and 2-[amino (3-chloroanilino)methylene]malononitrile, occurred in all the four comparisons under positive ionization mode (Figure 2; Supplementary Table S4).

#### 3.2.2 Hierarchical clustering analysis of all differential metabolites

Hierarchical clustering analysis was carried out on all the differential metabolites of the individual sample groups under positive (Supplementary Figure S1A) and negative (Supplementary Figure S1B) ionization mode. Under drought stress, *S. indica* inoculation resulted in great changes in metabolites levels in the needles of *P. taeda* seedlings. In detail, under positive ionization mode, some metabolites, such as taxifolin, hesperetin, indole-3-carboxylic acid, pteroside A, phenprobamate, 8-isoprostaglandin A2, celestolide, puerarin, demethylnobiletin, gardenin B, topotecan, stachydrine, uric acid, DL-panthenol, and vitamin A, showed higher levels in the needles of I_D seedlings, compared to their levels in the needles of NI_D seedlings (Supplementary Figure S2A). Under negative ionization mode, procyanidin B1, ixoside, nicotinamide adenine dinucleotide, toddalolactone, sibirioside A, hordatine A, tectoridin, tricin 5-*O*-hexoside, trifolirhizin, hesperetin 5-*O*-glucode, myricitrin, polydatin, calceolarioside B, artesunate, sattabacin, and casticin showed higher levels in the needles of I_D seedlings, compared to NI_D seedlings (Supplementary Figure S2B). Under positive ionization mode, the levels of methyl syringate, N-lauroylsarcosine, and corymboside increased in NI_D seedlings, increasing to 46.50, 34.45, and 23.03 time as those in NI_W seedlings, respectively (the file “NI_D.vs.NI_W_pos_Diff_order” in Supplementary Table S3). Under negative ionization mode, 8-iso-15-keto prostaglandin E2 increased to 39.50 times in NI_D seedlings as that in NI_W seedlings (the file “NI_D.vs._NI_W_neg_Diff_order” in Supplementary Table S3). Under positive ionization mode, eriocitrin, *trans*-aconitic acid, vitamin C, uric acid, alpha-ketoglutaric acid, vitamin A, stachydrine, and coumalic acid were upregulated more than three times in I_D seedlings, compared to their levels in NI_D seedlings (the file “I_D.vs.NI_D_pos_Diff_order” in Supplementary Table S3). Similarly, under negative ionization mode, itaconic acid, calceolarioside B, 2-oxoglutaric acid, and citric acid were upregulated more than three times in I_D seedlings, compared to their levels in NI_D seedlings (the file “I_D.vs.NI_D_neg_Diff_order” in Supplementary Table S3). Some of these differential metabolites showed close correlations (Supplementary material 2, including Supplementary Table S5), suggesting their synergetic roles in the needles of *P. taeda* seedlings under drought stress. All these results suggest that *S. indica* inoculation caused great differences in species and numbers of differential metabolites in *P. taeda* seedlings under drought stress.

#### 3.2.3 KEGG enrichment analysis

**Figure 3:**
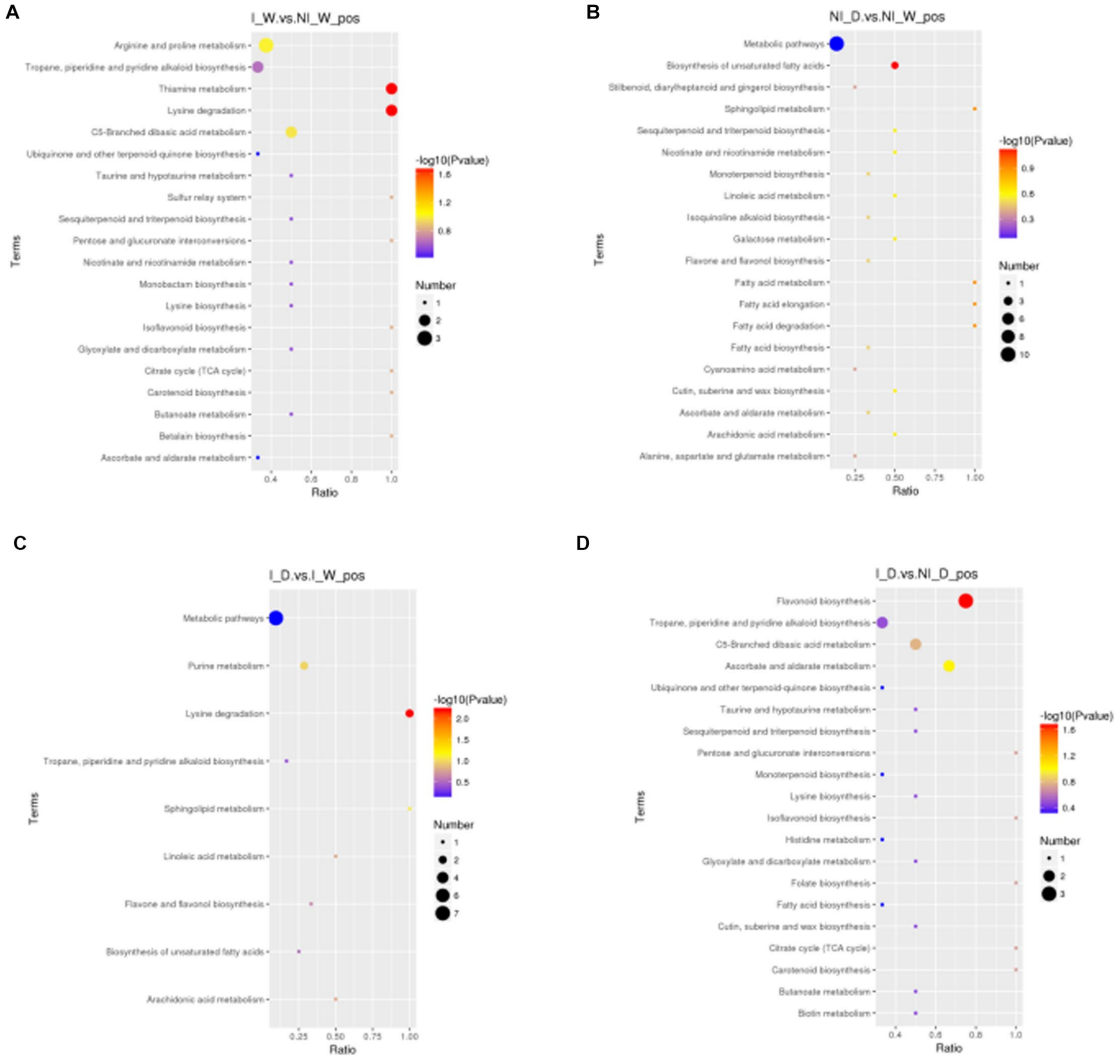
Metabolites enriched in KEGG pathways under positive ionization mode (top 20 pathways). In the small figures, horizontal axis represented the ratio of number of differential metabolites to the total numbers of metabolites identified in a pathway. The greater the ratio was, the higher the differential metabolites enriched in the pathway. The color of a circle represented value of *p* in geometric test. The size of a circle represented the number of differential metabolites in the corresponding pathway. **(A)** I_W vs. NI_W; **(B)** NI_D vs. NI_W; **(C)** I_D vs. I_W; **(D)** I_D vs. NI_D.I_W: inoculated seedlings under well-watered condition; NI_W: non-inoculated seedlings under well-watered condition; I_D: inoculated seedlings under drought stress; NI_D: non-inoculated seedlings under drought stress.

KEGG enrichment pathways are shown in Figure 3; Supplementary Figure S3; Supplementary Table S6. Under positive ionization mode, thiamine metabolism and lysine degradation were the most enriched in the comparison of I_W vs. NI_W (Figure 3A and the file “I_W.vs.NI_W_pos_kegg_enrichment” in Supplementary Table S6). In lysine degradation pathway, pipecolic acid and N6,N6,N6-trimethyl-L-lysine were enriched; in thiamine metabolism pathway, L-tyrosine and vitamin B1 were enriched (the file “I_W.vs.NI_W_pos_kegg_enrichment” in Supplementary Table S6). In the comparison, under negative ionization mode, the two pathways, namely, porphyrin and chlorophyll metabolism and tyrosine metabolism, were the most enriched (Supplementary Figure S3A and the file “I_W.vs.NI_W_neg_kegg_enrichment” in Supplementary Table S6). In porphyrin and chlorophyll metabolism, pheophorbide A and L-threonine were enriched; in tyrosine metabolism, homovanillic acid and levodopa were enriched (the file “I_W.vs.NI_W_neg_kegg_enrichment” in Supplementary Table S6).

In the comparison of NI_D vs. NI_W, six pathways were the most enriched under positive ionization mode, i.e., biosynthesis of unsaturated fatty acids, fatty acid elongation, fatty acid degradation, sphingolipid metabolism, fatty acid metabolism, and galactose metabolism. In these pathways, arachidonic acid, palmitic acid, 4-D-hydroxysphinganine, and raffinose were enriched (Figure 3B and the file “NI_D.vs.NI_W_pos_kegg_enrichmen” in Supplementary Table S6). In the comparison, under negative ionization mode, pyruvate metabolism was the most enriched (Supplementary Figure S3B and the file “NI_D.vs.NI_W_neg_kegg_enrichmen” in Supplementary Table S6). In the pathway, fumaric acid was enriched (the file “NI_D.vs.NI_W_neg_kegg_enrichmen” in Supplementary Table S6).

In the comparison of I_D vs. I_W, lysine degradation, sphingolipid metabolism, and purine metabolism were the most enriched pathways under positive ionization mode, and some differential metabolites, i.e., pipecolic acid, N6,N6,N6-trimethyl-L-lysine, 4-D-hydroxysphinganine, uric acid, and hypoxanthine, were enriched (Figure 3C and the file “I_D.vs.I_W_pos_kegg_enrichment” in Supplementary Table S6). In the comparison, under negative ionization mode, the three pathways, i.e., fructose and mannose metabolism, D-arginine and D-ornithine metabolism, and amino sugar and nucleotide sugar metabolism, were the most enriched (Supplementary Figure S3C and the file “I_D.vs.I_W_neg_kegg_enrichment” in Supplementary Table S6). In the three pathways, α-D-mannose 1-phosphate and L-ornithine were enriched (the file “I_D.vs.I_W_neg_kegg_enrichment” in Supplementary Table S6).

In the comparison of I_D vs. NI_D, the three pathways, i.e., flavonoid biosynthesis, ascorbate and aldarate metabolism, and C5-branched dibasic acid metabolism, were the most enriched under positive ionization mode (Figure 3D and the file “I_D.vs.NI_D_pos_kegg_enrichment” in Supplementary Table S6). In the three pathways, some differential metabolites were enriched, such as hesperetin, myricetin, taxifolin, alpha-ketoglutaric acid, vitamin C, and *trans*-aconitic acid (the file “I_D.vs.NI_D_pos_kegg_enrichment” in Supplementary Table S6). In the comparison, under negative ionization mode, the four pathways, i.e., thiamine metabolism, oxidative phosphorylation, phenylalanine, tyrosine, and tryptophan biosynthesis, and C5-branched dibasic acid metabolism, were the most enriched (Supplementary Figure S3D and the file “I_D.vs.NI_D_neg_kegg_enrichment” in Supplementary Table S6). Nicotinamide adenine dinucleotide, 5-dehydroquinic acid, and itaconic acid were enriched in these pathways under negative ionization mode (the file “I_D.vs.NI_D_neg_kegg_enrichment” in Supplementary Table S6).

### 3.3 Effects of *Serendipita indica* colonization on untargeted proteome

#### 3.3.1 Analysis on differential proteins

After function annotation of proteins and quantitative analysis (shown in Supplementary material 3, including Supplementary Tables S7, S8), differential proteins were analyzed. Statistic results of differential protein analysis showed that 32 and 14 differential proteins with FC > 2.0 were up- and downregulated, respectively, in the comparison of I_W vs. NI_W; 16 and 19 proteins were up- and downregulated, respectively, in the comparison of NI_D vs. NI_W; 13 and 21 proteins were up- and downregulated, respectively, in the comparison of I_D vs. I_W; 14 and 7 proteins were up- and downregulated, respectively, in the comparison of I_D vs. NI_D (Table 1; Supplementary Table S9).

**Table 1:** Statistic results of differential proteins.

Compared Samples	Num. of total quant.	Regulated type	FC > 1.2	FC > 1.3	FC > 1.5	FC > 2.0
I_W vs. NI_W	704	Upregulated	149	103	62	32
		Downregulated	66	53	35	14
NI_D vs. NI_W	701	Upregulated	64	51	32	16
		Downregulated	128	98	59	19
I_D vs. I_W	702	Upregulated	50	40	28	13
		Downregulated	114	87	48	21
I_D vs. NI_D	700	Upregulated	102	75	47	14
		Downregulated	24	17	13	7

**Figure 4:**
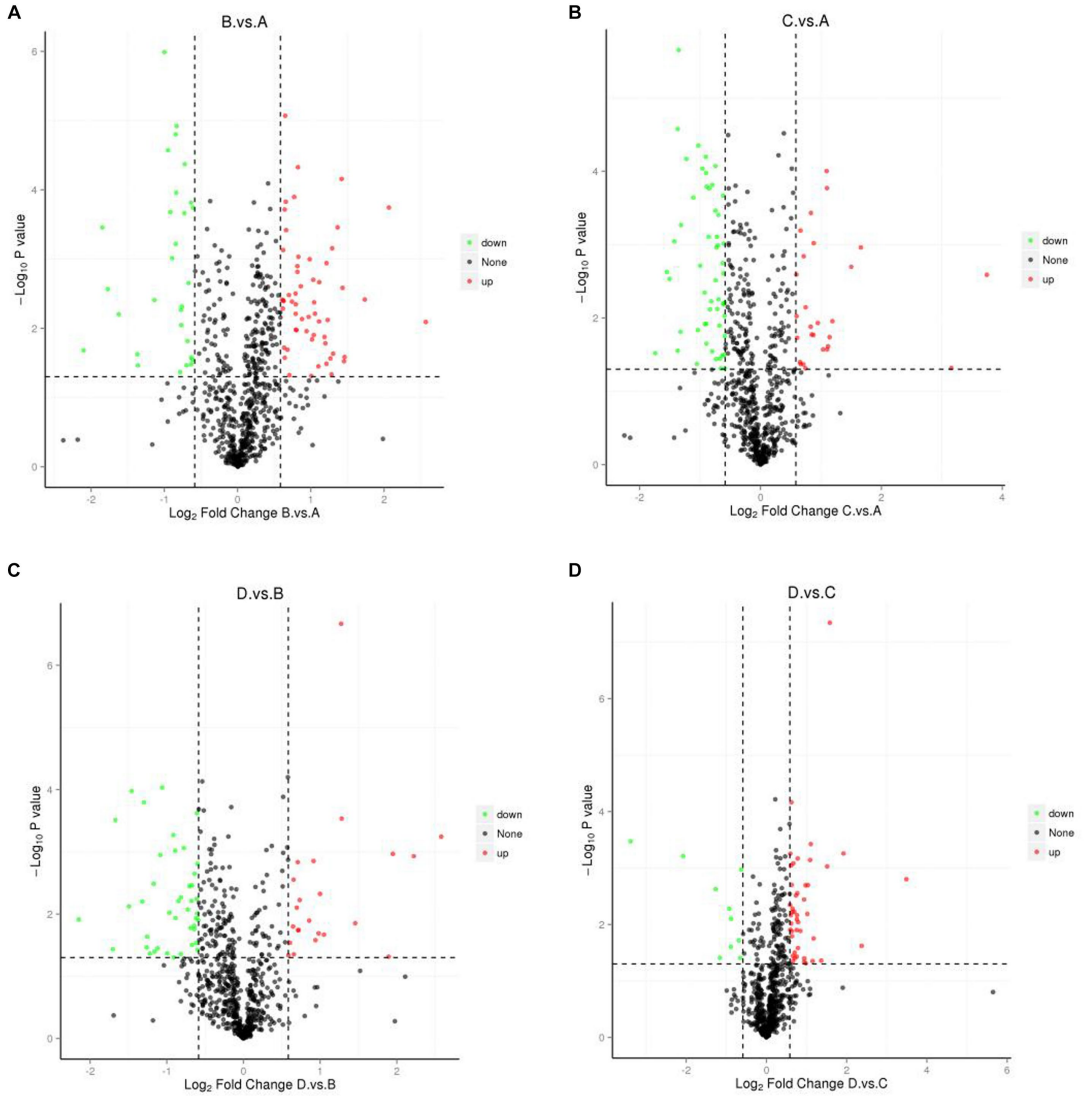
Volcano plots showing differential proteins. **(A)** I_W vs. NI_W; **(B)** NI_D vs. NI_W; **(C)** I_D vs. I_W; **(D)** I_D vs. NI_D. I_W: inoculated seedlings under well-watered condition; NI_W: non-inoculated seedlings under well-watered condition; I_D: inoculated seedlings under drought stress; NI_D: non-inoculated seedlings under drought stress.

Volcano plots showed changes in FCs of differential proteins under different comparisons (Figure 4), suggesting that some proteins were greatly affected by the four different treatments. Some proteins were greatly upregulated in the comparison of I_W vs. NI_W. For example, six proteins, i.e., A0A385JF21, H9VK00, H9V5W8, Q06IN7, K7NJV0, and H9WJS5, only occurred in the needles of I_W seedlings, and FCs of other three proteins (H9VRU0, A0A3G6JFD6, and Q84KL6) were more than 3.0 (the file “I_W.vs.NI_W.diff_prot” in Supplementary Table S10). At the same time, some proteins were greatly downregulated, such as R4L654, H9XAU6, H9W9Y6, H9X0G1, and H9WTG5 (the file “I_W.vs.NI_W.diff_prot” in Supplementary Table S10).

In the comparison of NI_D vs. NI_W, five proteins, i.e., H9V821, A0A385JF21, H9V5W8, K7NJV0, and H9WW19, only occurred in the needles of NI_D seedlings, and FCs of H9VCA2 and O22430 were more than 3.0 (the file “NI_D.vs.NI_W.diff_prot” in Supplementary Table S10). At the same time, some proteins, such as H9WTS5, H9X1G6, H9XAC0, R4L654, H9VRX6, H9W9Y6, H9VZM7, and H9WTG5, were greatly downregulated (the file “NI_D.vs.NI_W.diff_prot” in Supplementary Table S10).

In the comparison of I_D vs. I_W, five proteins, i.e., H9V821, A0A3G6JA21, K7NMB8, H9WSD7, and H9WKX6, only occurred in the needles of I_D seedlings, and FCs of H9VNE7, Q41096, H9X0G1, and O22430 were more than 3.0 (the file “I_D.vs.I_W.diff_prot” in Supplementary Table S10). At the same time, some proteins were greatly downregulated, such as H9VNX9, H9WAU3, A0A023SGT4, H9VRU0, and H9X861 (the file “I_D.vs.I_W.diff_prot” in Supplementary Table S10).

In the comparison of I_D vs. NI_D, two proteins, i.e., H9X056 and H9VDW5, only occurred in the needles of I_D seedlings, and FCs of H9VNE7, A0A385JF23, and H9W5R1 were more than 11.0, 5.0, and 3.0, respectively (the file “I_D.vs.NI_D.diff_prot” in Supplementary Table S10). At the same time, some proteins were greatly downregulated, such as H9WD94, H9WLL7, H9X861, and H9VCA2 (the file “I_D.vs.NI_D.diff_prot” in Supplementary Table S10).

#### 3.3.2 Analysis on enriched differential proteins

**Figure 5:**
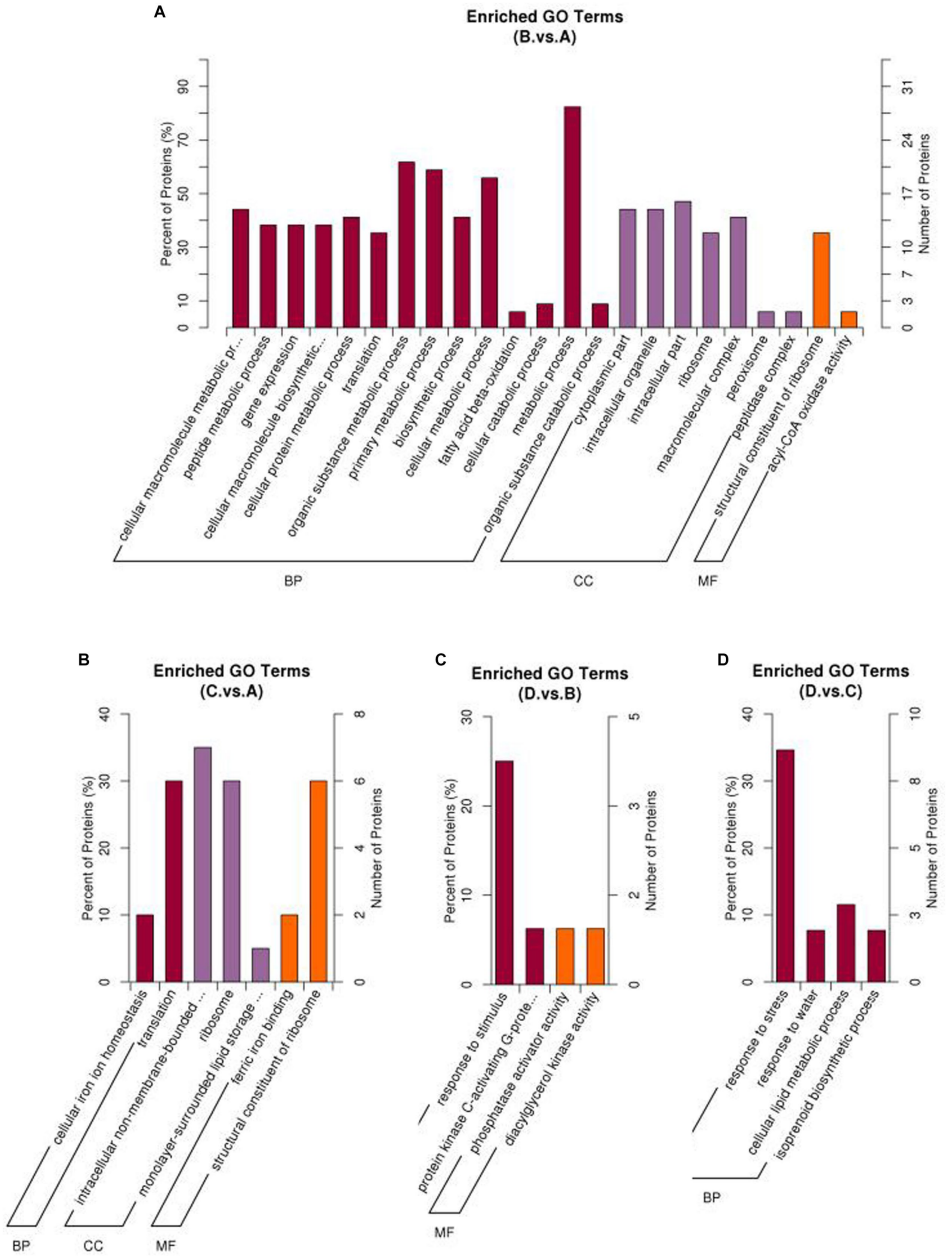
Percentage of upregulated proteins in different enriched GO terms under different comparisons. **(A)** I_W vs. NI_W; **(B)** NI_D vs. NI_W; **(C)** I_D vs. I_W; **(D)** I_D vs. NI_D. I_W: inoculated seedlings under well-watered condition; NI_W: non-inoculated seedlings under well-watered condition; I_D: inoculated seedlings under drought stress; NI_D: non-inoculated seedlings under drought stress.

GO enrichment analysis showed that different treatments greatly affected enrichment of differential proteins in different GO terms (Figure 5). In the comparison of I_W vs. NI_W, the most enriched GO term was “metabolic process” in the section of “Biological process.” In this GO term, 36 proteins were significantly regulated, with 28 and 8 proteins up- and downregulated, respectively (Figure 5A, the file “I_W.vs.NI_W.GO_Enrich.updown” in Supplementary Table S11). In the section of “Molecular Function,” the GO term “structural constituent of ribosome” was the most enriched (Figure 5A). In the GO term, 12 and 3 proteins (including R4LC26, R4L654, and H9X6V5) were up- and downregulated, respectively (the file “I_W.vs.NI_W.GO_Enrich.updown” in Supplementary Table S11).

In the other three comparisons, the numbers of enriched GO terms were small (Figures 5B–D). In the comparison of I_D vs. NI_D, the most enriched GO term was “response to stress” (Figure 5D). In the GO term, 9 and 1 proteins were up- and downregulated (Figure 5D, the file “I_D.vs.NI_D.GO_Enrich.updown” in Supplementary Table S11). In the GO term “response to water,” the two proteins (i.e., A0A2D1UKD2 and A0A023W8I6) were upregulated (Figure 5D, the file “I_D.vs.NI_D.GO_Enrich.updown” in Supplementary Table S11). Barplots of downregulated GO terms are shown in Supplementary Figure S4.

**Figure 6:**
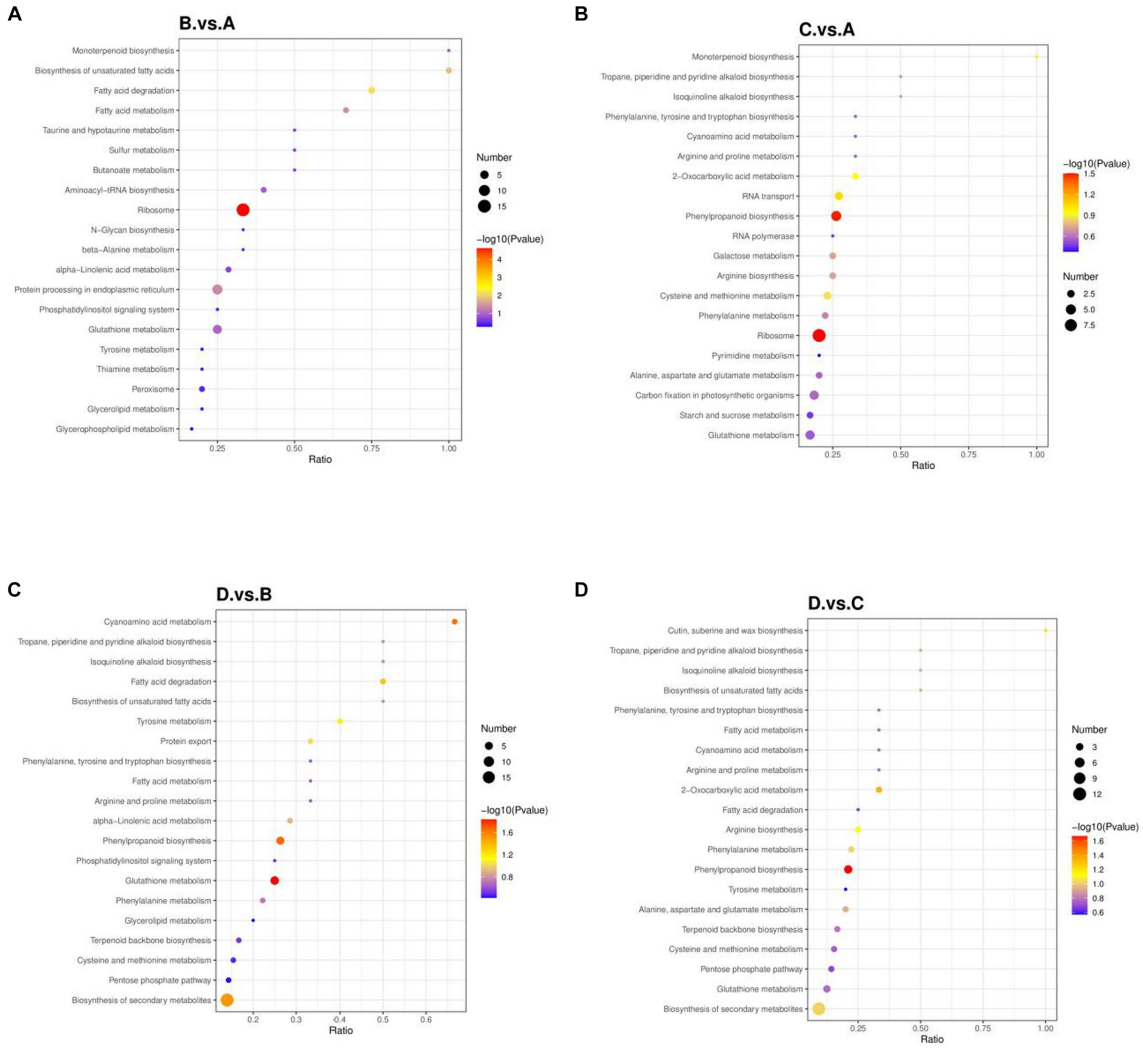
Bubble charts of KEGG pathways. **(A)** I_W vs. NI_W; **(B)** NI_D vs. NI_W; **(C)** I_D vs. I_W; **(D)** I_D vs. NI_D. In a bubble charts, *x* axis stands for the ratio of the number of differential proteins in a pathway to the total number of identified proteins in the pathway. The higher the ratio is, the higher the enriched differential proteins. The color of a round spot stands for value of *p* in the hypergeometric test. The color changes from blue to red. The smaller value of *p* is, the more reliable the hypergeometric test is, and the more significant the hypergeometric test is. The size of a round spot stands for the number of differential proteins in a pathway. The bigger the size of a round spot is, the more the number of differential proteins in a pathway is. I_W: inoculated seedlings under well-watered condition; NI_W: non-inoculated seedlings under well-watered condition; I_D: inoculated seedlings under drought stress; NI_D: non-inoculated seedlings under drought stress.

KEGG enrichment analysis is shown in Figure 6, and the top 20 enriched pathways were exhibited. In the comparison of I_W vs. NI_W, the most enriched pathways were monoterpenoid biosynthesis and biosynthesis of unsaturated fatty acids (Figure 6A). In the first pathway, the protein H9XB69 was enriched; in the second pathway, the two proteins, namely, H9W5R1 and H9V8G2, were enriched (the file “I_W.vs.NI_W.KEGG_Enrich” in Supplementary Table S12). In addition, ribosome pathway contained the greatest number of differential proteins, and the next were protein processing in endoplasmic reticulum and glutathione metabolism (Figure 6A). In the comparison of NI_D vs. NI_W, the most enriched pathways were isoquinoline alkaloid biosynthesis and tropane, piperidine, and pyridine alkaloid biosynthesis, and the protein H9X056 was involved in both of the two pathways (Figure 6B, the file “NI_D.vs.NI_W.KEGG_Enrich” in Supplementary Table S12). In the comparison of I_D vs. I_W, cyanoamino acid metabolism was the most enriched pathway (Figure 6C). In this pathway, two proteins, namely, H9XB10 and H9W1J3, were identified (the file “I_D vs. I_W.KEGG_Enrich” in Supplementary Table S12). In addition, biosynthesis of secondary metabolites contained the greatest number of identified differential proteins, and the next were phenylpropanoid biosynthesis and glutathione metabolism (Figure 6C). In the comparison of I_D vs. NI_D, the most enriched pathway was cutin, suberine, and wax biosynthesis (Figure 6D). In this pathway, the protein H9WHC3 was identified (the file “I_D.vs.NI_D.KEGG_Enrich” in Supplementary Table S12). Biosynthesis of secondary metabolites contained the greatest number of identified differential proteins, and the next was phenylpropanoid biosynthesis (Figure 6D).

**Figure 7:**
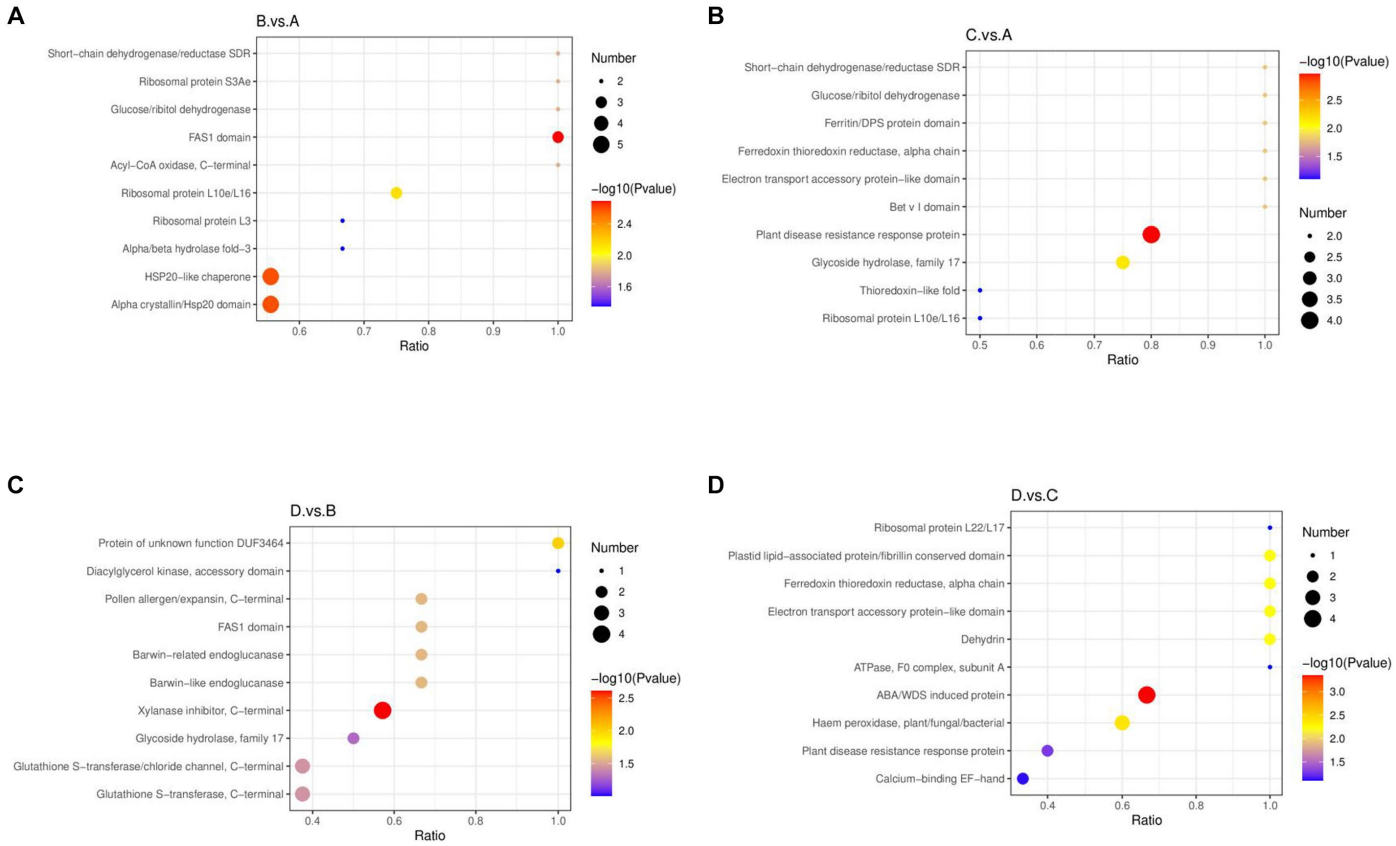
Bubble diagrams of enriched structural domains of differential proteins. **(A)** I_W vs. NI_W; **(B)** NI_D vs. NI_W; **(C)** I_D vs. I_W; **(D)** I_D vs. NI_D. In a bubble diagram, *x* axis stands for the ratio of the numbers of differential proteins with the responsive structural domain to the number of total number of identified proteins with the structural domain. The higher the ratio is, the more the number of differential proteins with the structural domain is. The color of a round spot stands for value of *p* in the hypergeometric test. The color changes from blue to red. The smaller value of *p* is, the more reliable the hypergeometric test is, and the more significant the hypergeometric test is. The size of a round spot stands for the number of proteins with the responsive structural domain. The bigger the size of a round spot is, the more the number of differential proteins with the structural domain is. I_W: inoculated seedlings under well-watered condition; NI_W: non-inoculated seedlings under well-watered condition; I_D: inoculated seedlings under drought stress; NI_D: non-inoculated seedlings under drought stress.

In aspect of structural domain enrichment, the four different treatments also affected the expression of differential proteins with different structural domains (Figure 7). In the comparison of I_W vs. NI_W, the proteins with the structural domains (short-chain dehydrogenase/reductase SDR, ribosomal protein S3Ae, glucose/ribitol dehydrogenase, FAS1 domain, and acyl-CoA oxidase) were enriched, and the two structural domains, such as HSP20-like chaperone and alpha crystallin/Hsp20 domain, contained the greatest numbers of identified differential proteins (Figure 7A, the file “I_W.vs.NI_W.IPR_Enrich.merge” in Supplementary Table S13). In the comparison of NI_D vs. NI_W, the proteins with these structural domains (short-chain dehydrogenase/reductase SDR, glucose/ribitol dehydrogenase, ferritin/DPS protein domain, ferredoxin thioredoxin reductase electron transport accessory protein-like domain, and bet v I domain) were enriched (Figure 7B), and the structural domain “plant disease resistance response protein” contained the greatest number of identified differential proteins (the file “NI_D.vs.NI_W.IPR_Enrich.merge” in Supplementary Table S13). In the comparison of I_D vs. I_W, the proteins with the two structural domains (protein of unknown function DUF3464 and diacylglycerol kinase, accessory domain) were enriched (Figure 7C). The structural domains, xylanase inhibitor (C-terminal), glutathione S-transferase/chloride channel (C-terminal), and glutathione S-transferase (C-terminal) contained more identified differential proteins (Figure 7C, the file “I_D.vs.I_W.IPR_Enrich.merge” in Supplementary Table S13). In the comparison of I_D vs. NI_D, the proteins with the structural domains, i.e., ribosomal protein L22/L17, plastid lipid-associated protein/fibrillin conserved domain, ferredoxin thioredoxin reductase (alpha chain), electron transport accessory protein-like domain, dehydrin, and ATPase (F0 complex, subunit A), were enriched (Figure 7D). In addition, the structural domain “ABA/WDS-induced protein” contained the greatest number of proteins (Q41087, Q41095, Q41096, and A0A023W8D4) (Figure 7D, the file “I_D.vs.NI_D.IPR_Enrich.merge” in Supplementary Table S13).

**Figure 8:**
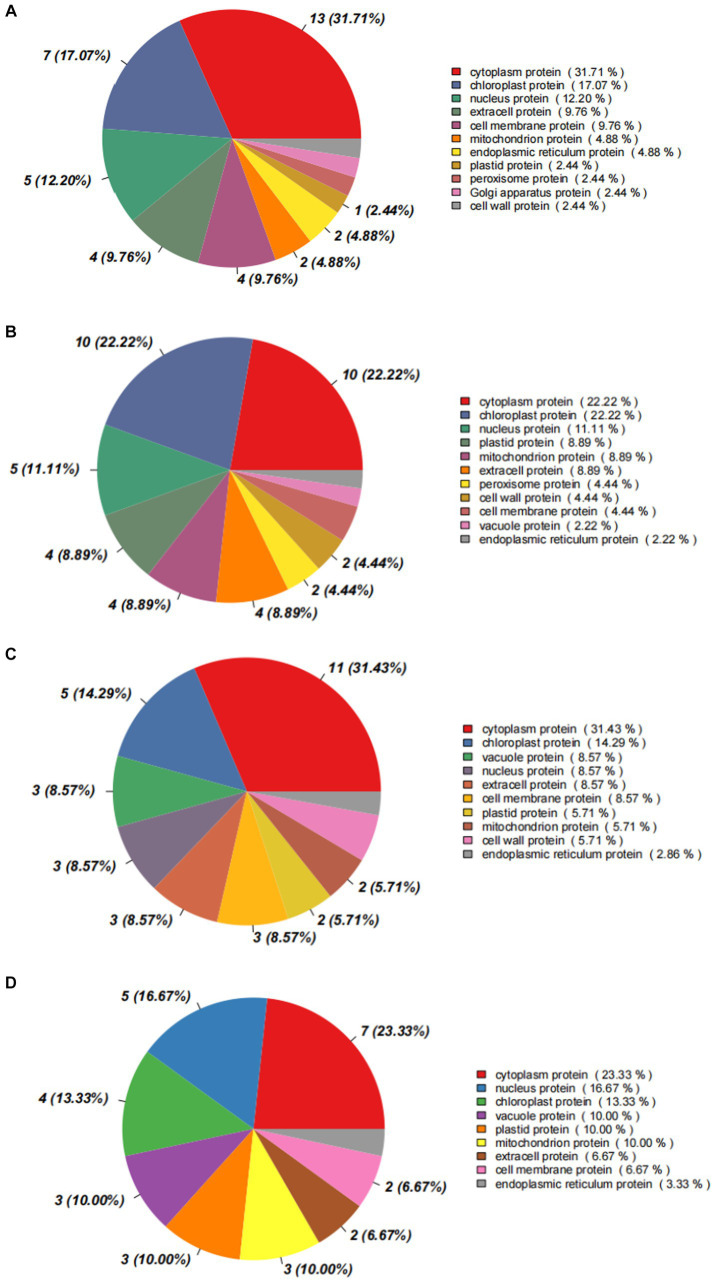
Statistic diagrams of subcellular localization of differential proteins. **(A)** I_W vs. NI_W; **(B)** NI_D vs. NI_W; **(C)** I_D vs. I_W; **(D)** I_D vs. NI_D. I_W: inoculated seedlings under well-watered condition; NI_W: non-inoculated seedlings under well-watered condition; I_D: inoculated seedlings under drought stress; NI_D: non-inoculated seedlings under drought stress.

The analysis on subcellular localization showed that differential proteins mainly distributed in cytoplasm (31.71%), chloroplasts (17.07%), and nuclei (12.20%), in the comparison of I_W vs. NI_W, together occupying more 60% of the total number (Figure 8A). In chloroplasts, H9X1G6 and H9VZM7 are SHSP domain-containing proteins; in cytoplasm, most differential proteins were uncharacterized; and in nuclei, H9V4J8 and H9VFX4 are also SHSP domain-containing proteins (the file “I_W.vs.NI_W.Subcellular_diff” in Supplementary Table S14). In the comparison of NI_D vs. NI_W, most differential proteins also mainly distributed cytoplasm (22.22%), chloroplasts (22.22%), and nuclei (11.11%) (Figure 8B). In chloroplasts, H9VCA2 and H9VCA1 are two FeThred_A domain-containing proteins, and H9X1G6 and H9VZM7 are two SHSP domain-containing proteins; in cytoplasm, H9WKD5 and H9WFS7 are two Bet_v_1 domain-containing proteins; in nuclei, two proteins, namely, Q41095 and Q41096, are water deficit-inducible, encoded by the genes *lp3-3* and *lp3-2*, respectively (the file “NI_D.vs.NI_W.Subcellular_diff” in Supplementary Table S14). In the comparison of I_D vs. I_W, differential proteins mainly distributed in cytoplasm (31.43%), chloroplasts (4.29%), and vacuoles (8.57%) (Figure 8C). Some new proteins occurred in cytoplasm, chloroplasts, and vacuoles, such as H9W1G7 (SHSP domain-containing protein) in cytoplasm, A5HIY3 (thaumatin-like protein) in vacuoles, Q84KL6 (alpha-pine synthase) in chloroplasts, and O24314 and Q41096 (water deficit-inducible protein) in nuclei (the file “I_D.vs.I_W.Subcellular_diff” in Supplementary Table S14). In the comparison of I_D vs. NI_D, differential proteins also distributed in cytoplasm (23.33%), nuclei (16.67%), and chloroplasts (13.33%) (Figure 8D). More notably, four water deficit-inducible proteins, i.e., Q41087, Q41095, Q41096, and A0A023W8D4, occurred in nuclei (the file “I_D.vs.NI_D.Subcellular_diff” in Supplementary Table S14).

#### 3.3.3 Interactions between differential proteins

**Figure 9:**
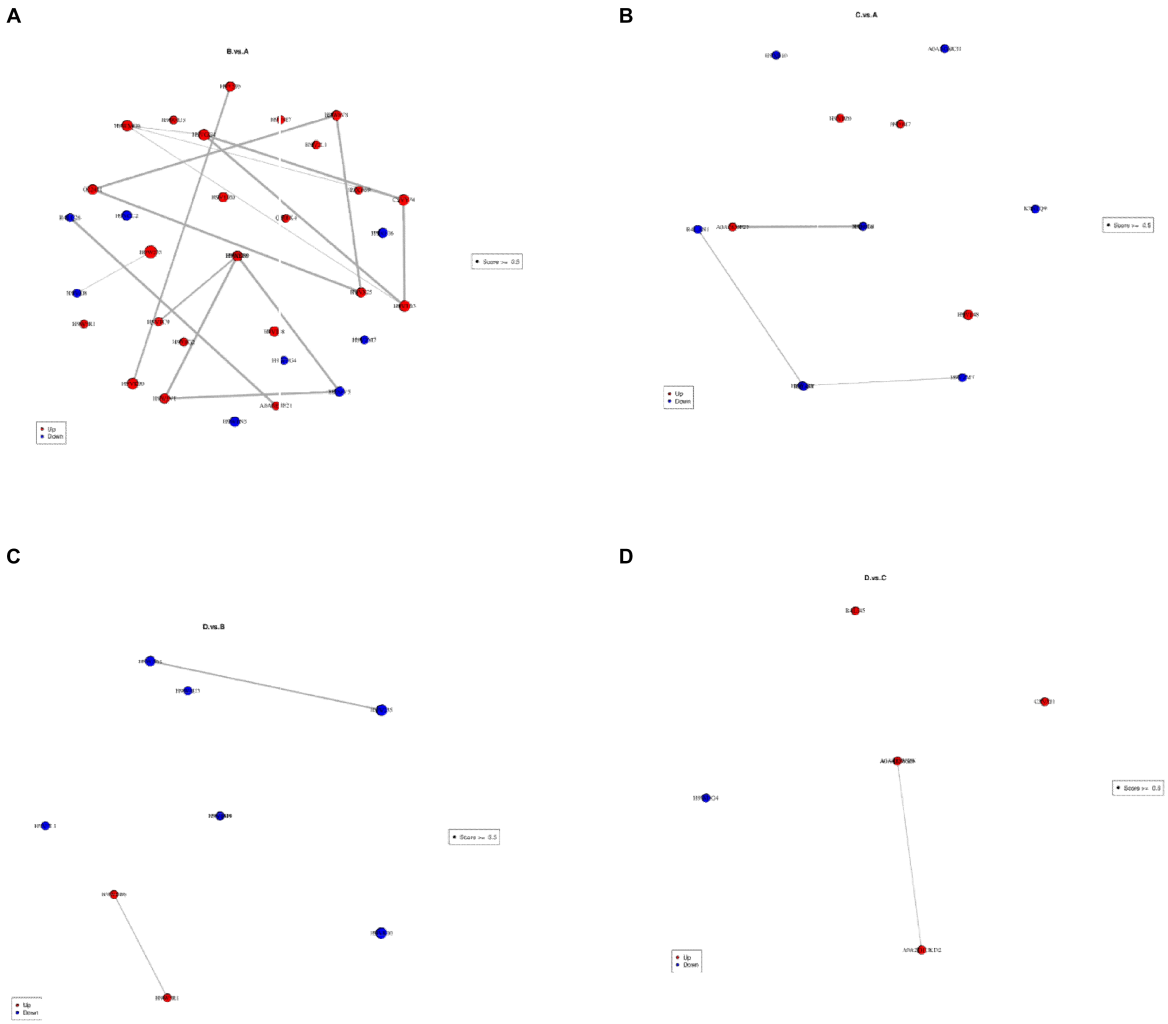
Analysis on interactions between differential proteins. In each diagram, each spot stands for a differential protein labeled with its name. **(A)**: I_W vs. NI_W; **(B)**: NI_D vs. NI_W; **(C)**: I_D vs. I_W; **(D)**: I_D vs. NI_D. I_W: inoculated seedlings under well-watered condition; NI_W: non-inoculated seedlings under well-watered condition; I_D: inoculated seedlings under drought stress; NI_D: non-inoculated seedlings under drought stress.

To investigate the interactions between differential proteins, StringDB data bank^2^ was used to analyze the interactions (Figure 9; Supplementary Table S15). Under the comparison of I_W vs. NI_W, strong interactions occurred between significantly upregulated proteins, such as the protein O22431 and the two proteins H9VX25 and H9W8V8, H9V595 and H9VK00, H9VGG4 and C3VYP4, H9VLB3 and C3VYP4, H9VX25 and H9W8V8, and H9WJV1 and H9WG89 (Figure 9A; Supplementary Table S15). In the comparison of NI_D vs. NI_W, weak interactions occurred between significantly downregulated proteins, such as H9W129 and H9VZM7, and R4LAN1 and R4L654 (Figure 9B; Supplementary Table S15). In the comparison of I_D vs. I_W, strong interactions occurred between significantly downregulated proteins, such as H9V4M9 and H9VGG4, and H9WN61 and H9W235; strong interaction occurred between significantly upregulated proteins, i.e., H9W5R1 and H9WBB6 (Figure 9C; Supplementary Table S15). In the comparison of I_D vs. NI_D, strong interaction only occurred between the significantly upregulated proteins, i.e., A0A023W8I6 and A0A2D1UKD2 (Figure 9D; Supplementary Table S15).

### 3.4 Correlations between differential metabolites and proteins

**Figure 10:**
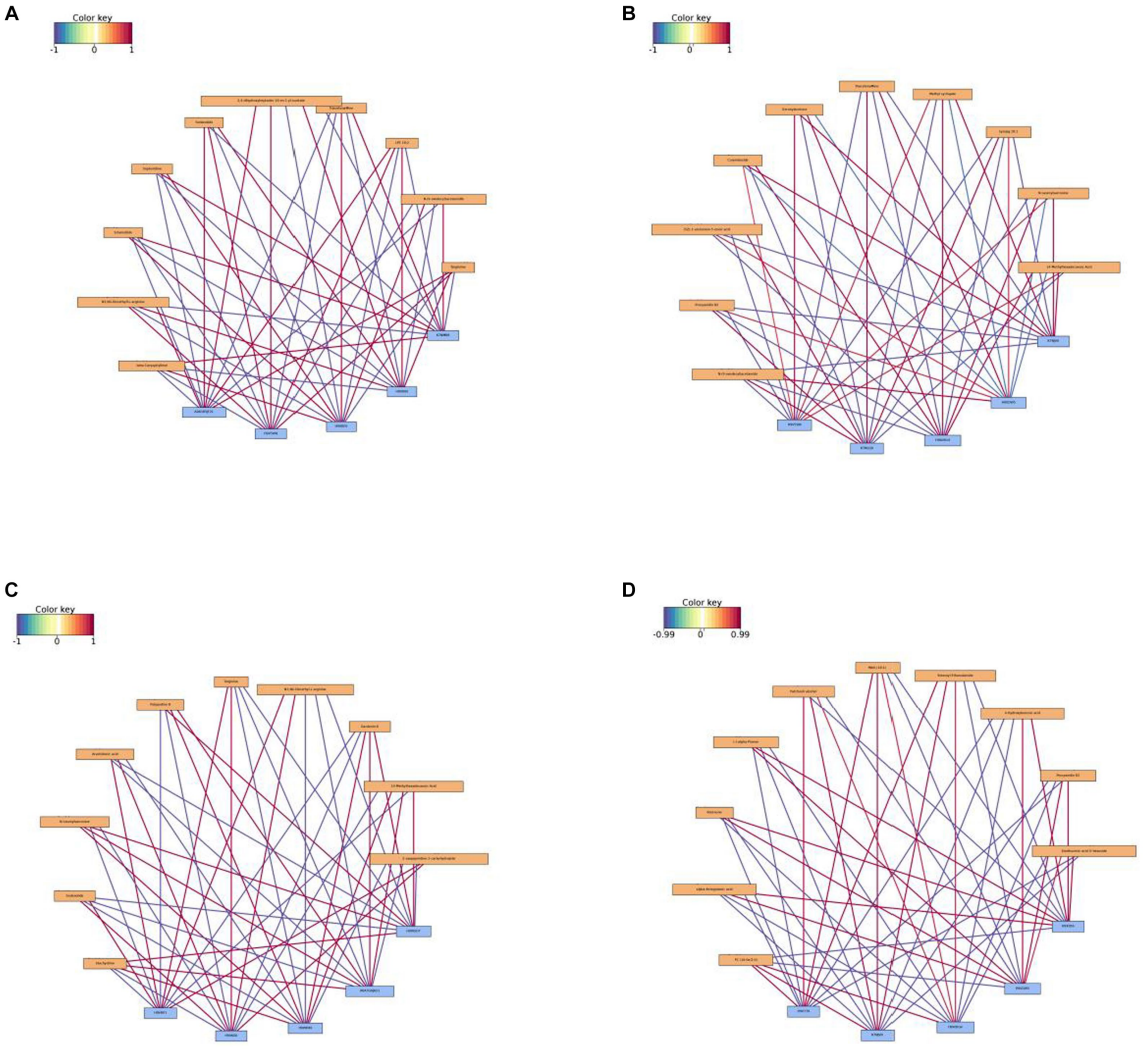
Network diagrams of correlation analysis under positive ionization mode. **(A)** I_W vs. NI_W; **(B)** NI_D vs. NI_W; **(C)** I_D vs. I_W; **(D)** I_D vs. NI_D. The top 10 differential proteins and the top 5 differential metabolites were used in correlation analysis. In a network diagram, yellow stands for a differential metabolite, and blue stands for a differential protein. Red line stands for positive correlation, and blue line stands for negative correlation. The deeper the color is, the bigger the correlation coefficient is. I_W: inoculated seedlings under well-watered condition; NI_W: non-inoculated seedlings under well-watered condition; I_D: inoculated seedlings under drought stress; NI_D: non-inoculated seedlings under drought stress.

Correlations between differential metabolites and proteins were analyzed (Figure 10). In the comparison of I_W vs. NI_W, under positive ionization mode, the protein H9V5W8 was in significantly positive correlations to few metabolites, including targinine, LPE 18:2, 2,4-dihydroxyheptadec-16-3n-1-yl acetate, sedanolide, and N3,N4-dimethyl-L-arginine; the protein K7NMB8 was in significantly positive correlation to few metabolites, such as beta-caryophyllene, sclareolide, N-(9-oxodecyl)acetamide, sophoridine, and triacetonamine (Figure 10A and the file “I_W.vs.NI_W_pos_I_W.vs.NI_W_corr” in Supplementary Table S16). In addition, proline biosynthesis was in significantly positive correlations to some proteins, such as the three proteins, namely, A0A023SGT4, A0A385JF21, and A0A3G6JDK7, but in significantly negative correlations to some proteins, such as the four proteins H9V143, H9V4J8, H9V5X1, and H9VCC2 (the file “I_W.vs.NI_W_pos_I_W.vs.NI_W_corr” in Supplementary Table S16). Under negative ionization mode, gibberellic acid was in significantly positive correlations to the three proteins, i.e., A0A385JF21, H9V5W8, and H9VK00, but in significantly negative correlations to the two proteins, i.e., K7NMB8 and H9XBF0 (Supplementary Figure S5A and the file “I_W.vs.NI_W_neg_I_W.vs.NI_W_corr” in Supplementary Table S16). Salicylic acid *O*-glucoside was in significantly positive correlations to the four proteins, A0A385JF21, H9V5W8, H9VBI7, and H9VK00, but in significantly negative correlations to the three proteins, K7NMB8, H9V4J8, and H9XBF0 (Supplementary Figure S5A and the file “I_W.vs.NI_W_neg_I_W.vs.NI_W_corr” in Supplementary Table S16).

In the comparison of NI_D vs. NI_W under positive ionization mode, the metabolite methyl syringate was in significantly positive correlations to the three proteins, K7NJV0, H9WW19, and H9V5WB, but in significantly negative correlations to the two proteins, H9VDW5 and K7NLQ9; the metabolite N-lauroylsarcosine was in significantly positive correlations to the two proteins, K7NJV0 and H9WW19, but in significantly negative correlations to the two proteins, H9VDW5 and K7NLQ9 (Figure 10B and the file “NI_D.vs.NI_W_pos_NI_D.vs.NI_W_c” in Supplementary Table S16). In addition, vitamin C biosynthesis was in significantly positive correlations to the three proteins, A0A3G6J9T6, A0A3G6JA21, and A0A3G6JAC7, but in significantly negative correlations to the three proteins, A0A385JF21, B2CBS5, and H9V821 (the file “NI_D.vs.NI_W_pos_NI_D.vs.NI_W_c” in Supplementary Table S16). Under negative ionization mode, 4-coumaric acid was in significantly positive correlations to the three proteins, K7NJV0, H9WW19, and H9V5W8, but in significantly negative correlations to the two proteins, H9VDW5 and K7NLQ9 (Supplementary Figure S5B and the file “NI_D.vs.NI_W_neg_NI_D.vs.NI_W_c” in Supplementary Table S16). In addition, methyl jasmonate was in significantly positive correlations to the three proteins, A0A2D1UKD2, A0A385JEN6, and A0A3G6JA21, but in significantly negative correlations to the three proteins, A0A385JF21, H9V5W8, and H9V821 (the file “NI_D.vs.NI_W_neg_NI_D.vs.NI_W_c” in Supplementary Table S16).

In the comparison of I_D vs. I_W under positive ionization mode, both of stachydrine and N-lauroylsarcosine were in significantly positive correlations to the three proteins, H9WSD7, A0A3G6JA21, and H9WKX6, but in significantly negative correlations to the two proteins, H9VK00 and H9VRP1 (Figure 10C and the file “I_D.vs.I_W_pos_I_D.vs.I_W_corr” in Supplementary Table S16). The metabolite arachidonic acid just showed contrary correlations to these proteins (Figure 10C). Under negative ionization mode, L-ornithine was in significantly positive correlations to the two proteins, H9VRP1 and H9VK00, but in significantly negative correlations to the three proteins, H9WKX6, A0A3G6JA21, and H9WSD7; trifolirhizin was in significantly positive correlations to the three proteins, H9WSD7, A0A3G6JA21, and H9WKX6, but in significantly negative correlations to the two proteins, H9VRP1 and H9VK00 (Supplementary Figure S5C and the file “I_D.vs.I_W_neg_I_D.vs.I_W_corr” in Supplementary Table S16). In addition, methyl jasmonate was in significantly positive correlations to the three proteins, A0A023SGT4, A0A3G6JAC7, and A0A3G6JDJ9, but in significantly negative correlations to the three proteins, A0A023W8I6, A0A3G6JA21, and C3VXI1 (the file “I_D.vs.I_W_neg_I_D.vs.I_W_corr” in Supplementary Table S16).

In the comparison of I_D vs. NI_D under positive ionization mode, fatty acids PC (16:0e/2:0) and MAG (18:1) were in significantly positive correlations to the three proteins, H9V739, K7NJV0, and H9WDG4, but in significantly negative correlations to the two proteins, H9X056 and H9VDW5 (Figure 10D and the file “I_D.vs.NI_D_pos_I_D.vs.NI_D_cor” in Supplementary Table S16). The metabolite rotenone just showed the contrary correlations to these proteins (Figure 10D). In addition, vitamin C was in significantly positive correlations to the four proteins, A0A023W8D4, H9V6D7, A0A2D1UL07, and H9V5W8, but in significantly negative correlations to the three proteins, H9V739, H9VCA2, and H9VVX5 (the file “I_D.vs.NI_D_pos_I_D.vs.NI_D_cor” in Supplementary Table S16). Under negative ionization mode, the metabolites 4-coumaric acid and LPC18:0 were in significantly positive correlations to the three proteins, H9V739, K7NJV0, and H9WDG4, but in significantly negative correlations to the two proteins, H9X056 and H9VDW5 (Supplementary Figure S5B and the file “I_D.vs.NI_D_neg_I_D.vs.NI_D_cor” in Supplementary Table S16). The metabolite trifolirhizin just showed the contrary correlations to these proteins (Supplementary Figure S5D). In addition, the metabolite itaconic acid was in significantly positive correlations to the four proteins, A0A023W8I6, A0A023W8D4, C3VXI1, and C6ZG55, but in significantly negative correlations to the four proteins, H9V739, H9VCA2, H9VCR5, and H9WDG4 (the file “I_D.vs.NI_D_neg_I_D.vs.NI_D_cor” in Supplementary Table S16).

## 4 Discussion

As functions of symbiosis of mycorrhizal fungi (Chandrasekaran, 2022; Begum et al., 2023; Zou et al., 2023), plant growth-promoting rhizobacteria (PGPR) (Shankar and Prasad, 2023; Tanveer et al., 2023; Zhao et al., 2023), and dark septate endophytes (DSE) (Li et al., 2022; He C. et al., 2022; Liu et al., 2023) during drought stress, root endophytic fungi also play important roles in increasing tolerance to drought stress of their host plants (Azevedo et al., 2021; Manzur et al., 2022; Zuo et al., 2022). *S. indica* is also a root endophytic fungus, and it improves drought tolerance of their host plants, as described above. However, the related mechanisms are not clear. In this present study, combination of metabolomics and proteomics revealed the potential mechanisms.

### 4.1 *Serendipita indica*-induced drought tolerance by improving biosynthesis of flavonoids in *Pinus taeda*

Under drought stress, *S. indica* inoculation resulted in great changes in species and levels of flavonoids and their derivatives, such as taxifolin, hesperetin 5-*O*-glucode, puerarin, 5-demethylnobiletin, gardenin B, procyanidin B1, tectoridin, tricin, trifolirhizin, myricitrin, and eriocitrin (Supplementary Figures S1, S2; Supplementary Table S3). The metabolite eriocitrin (a dietary flavonoid) was the most enriched compound in the needles of I_D seedlings, increasing 4.86 times as that in the needles of NI_D seedlings, even more than the level of ascorbate (vitamin C, increasing 4.48 times as that in the needles of NI_D seedlings) (the file “I_D.vs.NI_D_pos_Diff_order” in Supplementary Table S3). Eriocitrin is an abundant flavonoid in lemons, which is known as a strong antioxidant agent (Kwon and Choi, 2020). Although it remain unclear about its functions in plants, especially under drought stress, the increased levels caused by *S. indica* inoculation with *P. taeda* seedlings should be related to its strong ability to scavenge reactive oxygen species (ROS) as an antioxidant. Its accurate function should be focused on in plants under drought stress. *S. indica* inoculation increased the level of taxifolin under drought stress (Supplementary Figure S3A; Supplementary Table S3). Taxifolin, i.e., dihydroquercetin, is a unique bioactive flavonoid. However, it is not clear about its functions in plants, especially under drought stress. But at present, it was known well about functions of quercetin in plants (Singh et al., 2021). The metabolite facilitates several plant physiological processes, such as seed germination, pollen growth, antioxidant machinery, and photosynthesis, as well as induces proper plant growth and development. Quercetin level increased in leaves and flowers of St. John’s wort (*Hypericum perforatum*) under drought stress (Gray et al., 2003). Since quercetin, a known flavonoid, acts as an antioxidant (Di Petrillo et al., 2022), it should be able to play an important role in scavenging ROS produced in plants under drought stress. More importantly, quercetin mediates ABA signaling (Singh et al., 2021); thus, the metabolite is involved in drought tolerance. Taxifolin is a derivative of quercetin; thus, its increased level caused by *S. indica* inoculation suggests that the metabolite might possess a more impalpable role in plants under drought stress, just like quercetin. In addition to taxifolin, the levels of two derivatives of quercetin increased in the needles of I_D seedlings, i.e., quercetin-3-O-beta-glucopyranosyl-6′-acetate (C_23_H_22_O_13_, the file “I_D.vs.NI_D_neg_Diff_order” in Supplementary Table S3) and quercetin-3-ramnoside (C_21_H_20_O_11_, the file “I_D.vs.NI_D_pos_Diff_order” in Supplementary Table S3), increasing 2.79 and 1.72 times, respectively. On the other hand, a report showed that the levels of some derivatives of quercetin reduced in the leaves of *Quercus pubescens* under drought stress, such as quercetin-3-O-glucose, quercetin pentose hexose, quercetin galloyl glucose, and quercetinhexose 1 (Saunier et al., 2022). Their levels reduced 36, 50, 45, and 33%, respectively. Altogether, the functions of quercetin derivatives are distinct from each other in plants under drought stress. Since the level of taxifolin increased in the needles of I_D seedlings, the increase should be useful for increased tolerance of *P. taeda* seedlings to drought stress.

*S. indica* inoculation also increased hesperetin level in the needles of *P. taeda* seedlings under drought stress (Supplementary Figure S3A), increasing 1.83 times, compared to its level in the needles of NI_D seedlings (the file “I_D.vs.NI_D_pos_Diff_order” in Supplementary Table S3). Hesperetin is a citrus flavonoid; however, no report showed its function in plants. Since it acts as an antioxidant in human cells (Famurewa et al., 2022), it is reasonable to speculate that it can scavenge free radicals in plants under drought stress or maybe it plays a specific role in plants under drought stress. Similarly, puerarin, an isoflavone, increased in the I_D seedlings (Supplementary Figure S3A), increasing 1.64 times as that in the needles of NI_D seedlings (the file “I_D.vs.NI_D_pos_Diff_order” in Supplementary Table S3). At present, it is not clear about its functions in plants under drought stress.

In the flavonoid biosynthesis pathway, the protein, H9WXL2, was enriched in the needles of I_D seedlings (the file “I_D.vs.NI_D.KEGG_Enrich” in Supplementary Table S12). H9WXL2 is NmrA domain-containing protein^3^. Those NmrA domain-containing proteins possess multiple functions in organisms. For example, in the soil-living protozoan, *Dictyostelium discoideum*, a protein with an NmrA-like domain, is required for cell differentiation and development (Núñez-Corcuera et al., 2008). In fungi, such proteins act as transcription repressors (Nichols et al., 2001; Zhao et al., 2010) and are involved in the post-translational modulation of the GATA-type transcription factor AreA, forming part of a system controlling nitrogen metabolite repression and stress resistance in various fungi (Stammers et al., 2001; Li et al., 2021). At present, it is not clear about the functions of NmrA domain-containing proteins in plants. Since *S. indica* inoculation improved H9WXL2 enrichment in needles of *P. taeda* seedlings under drought stress, the protein should possess its functions in *P. taeda* under drought stress. As mentioned above, the levels of many flavonoids increased in I_D seedlings, and H9WXL2 enrichment should be in positive correlation with these increased flavonoids.

### 4.2 *Serendipita indica*-induced drought tolerance by improving biosynthesis of organic acids in *Pinus taeda*

The levels of many organic acids increase in plants under drought stress and play important roles (Khosravi-Nejad et al., 2022; Ma et al., 2022; Wu et al., 2023). *S. indica* inoculation further increased biosynthesis of some organic acids in *P. taeda* seedlings under drought stress. In the needles of I_D seedlings, the levels of many organic acids are increased, compared to their levels in the needles of NI_D seedlings (the file “I_D.vs.NI_D_pos_Diff_order” in Supplementary Table S3). Out of them, *trans*-aconitic acid showed the highest increased level in I_D seedlings, 4.57 times as that of in NI_D seedlings (the file “I_D.vs.NI_D_pos_Diff_order” in Supplementary Table S3).

*Trans*-aconitic acid is a tricarboxylic acid and natural geometric isomer of *cis*-aconitic acid which is an intermediate in the citric acid cycle and with a well-defined role in energy metabolism. *Trans*-aconitic acid accumulated in surprisingly high concentrations in early-season forage grasses, such as *Hordeum leporinum* (3.5% of dry material), *Phalaris tuberosa* var. *stenoptera* (4.2% of dry material), and *Delphinium hesperium* (12.2% of dry leaves) (Burau and Stout, 1965). Burau and Stout (1965) speculated such high accumulation of *trans*-aconitic acid may be partially responsible for nutritional disorders, such as grass tetany (hypomagnesemia). As an allelochemical, *trans*-aconitic acid can reduce the germination and seed bank of weeds in the soil, and exogenous *trans*-aconitic acid applied in soil inhibits the growth and photosynthesis of *Glycine max* (Bortolo et al., 2018). It might be speculated that increased accumulation in the needles of I_D seedlings is related to inhibition of growth and photosynthesis. On the other hand, *trans*-aconitic acid acts as an inhibitor of glutamate decarboxylase (Endo and Kitahara, 1977), which catalyzes conversion of L-glutamate to aminobutanoate. This inhibition caused by the increased level of *trans*-aconitic acid might maintain well function of L-glutamate in I_D seedlings. *Trans*-aconitic acid is synthesized via potassium-dependent citrate dehydrase in maize (*Zea mays*), and its isozymes CD I and CD II are situated in mitochondria and cytosol, respectively (Brauer and Teel, 1982). It was reported that *trans*-aconitic biosynthesis by CD I is regulated by both citrate concentration and pH and that its biosynthesis by CD II is regulated by potassium (Brauer and Teel, 1982). Thus, the positive correlation between leaf potassium/citrate and *trans*-aconitic acid in maize was established. Therefore, increase in the level of *trans*-aconitic acid in the needles of I_D seedlings might suggest that high level of potassium ions/citrate occurred in these needles. Potassium ions possess strong physiological functions, especially under abiotic stress (Anil Kumar et al., 2022). The availability of potassium ions during drought stress increases the stomatal conductance with subsequent increase in water level (Oddo et al., 2020). Proline biosynthesis triggered by potassium ions has been found to be associated with osmotic adjustment and involved in scavenging ROS under drought stress (Jatav et al., 2012; Hasanuzzaman et al., 2018). Altogether, based the three aspects about *trans*-aconitic acid mentioned above, it is still difficult to explain the accurate function of *trans*-aconitic acid in inoculated *P. taeda* seedlings, and more experiments are necessary.

Next, to *trans*-aconitic acid, uric acid increased 4.22 times in the needles of I_D seedlings, compared to its level in NI_D seedlings (the file “I_D.vs.NI_D_pos_Diff_order” in Supplementary Table S3). Interestingly, uric acid deposits occurred in symbiotic marine algae when the marine algae symbiosed with *Aiptasia* sp. anemones (Clode et al., 2009), and uric acid deposits are likely to be associated with the nitrogen metabolism of the symbiosis. Exogenous application of uric acid enhanced the resistance of apple (*Malus*×*domestica* Borkh.) plants to salinity stress, and overexpression of MdNAT7 (nucleobase-ascorbate transporter, NAT) in apple plants enhanced uric acid concentrations and improved tolerance to salinity stress, compared with non-transgenic plants, while opposite phenotypes were observed for MdNAT7 RNAi plants (Sun et al., 2021). Therefore, it is possible to speculate that accumulation of uric acid in the needles of I_D seedlings might show two physiological functions to increase drought tolerance: scavenging ROS and involvement in nitrogen metabolism.

In the needles of I_D seedlings, the level of alpha-ketoglutaric acid increased to 3.93 times as that in NI_D seedlings (the file “I_D.vs.NI_D_pos_Diff_order” in Supplementary Table S3). The increase is helpful for improvement of drought tolerance of *P. taeda* seedlings, because foliar application of alpha-ketoglutarate improves drought resistance in soybean (*Glycine max*) (Gai et al., 2022). Exogenous alpha-ketoglutarate significantly increased proline concentrations in the leaves of the two soybean cultivars (drought resistant “Henong 51” and drought-sensitive “Henong43”) (Gai et al., 2022). In needles of I_D seedlings, the level of coumalic acid increased to 3.23 times as that in NI_D seedlings (the file “I_D.vs.NI_D_pos_Diff_order” in Supplementary Table S3). Information about functions of coumalic acid in plants was known less; therefore, it is difficult to explain why *S. indica* inoculation increased the level of coumalic acid in the needles of *P. taeda* seedlings under drought stress.

Under drought stress, stachydrine increased to 3.26 times in the needles of inoculated seedlings as that in non-inoculated seedlings under drought stress (the file “I_D.vs.NI_D_pos_Diff_order” in Supplementary Table S3). Stachydrine (alias proline betaine) is an osmoprotectant that is at least as effective as glycine betaine and more effective than L-proline, for various strains of *Staphylococcus aureus*, *Staphylococcus epidermidis*, and *Staphylococcus saprophyticus* (Amin et al., 1995). It was reported that symbiosis between arbuscular mycorrhizal fungus *Glomus intraradices* and sugarcane plantlets (*Saccharum* spp. cv. Mex 69–290) increased the levels of proline and glycine betaine under drought stress (Spinoso-Castillo et al., 2023). Therefore, the increased level of stachydrine caused by *S. indica* inoculation is helpful for *P. taeda* seedlings under drought stress. Vitamin C increased in the needles of I_D seedlings, compared to those of NI_D seedlings (the file “I_D.vs.NI_D_pos_Diff_order” in Supplementary Table S3). Ascorbic acid (vitamin C) is a strong antioxidant and plays important roles in plants under abiotic stress.

Under negative ionization mode, itaconic acid was detected to increase to more than three times in I_D seedlings as that of NI_D seedlings (Supplementary Table S3). Itaconic acid and its derivatives possess functions via affecting plant growth and development. Exogenous application of the itaconic acid derivative, hexylitaconic acid (100 mg·L^−1^), promoted the growth of rice seedlings by 20–30%, indicating that the derivative shows plant growth-regulating activity (Isogai et al., 1984). However, the three derivative of itaconic acid, i.e., octyl itaconic acid, deoxysporothric acid, and epideoxysporothric acid, inhibited radicle and germ growth of the dicotyledon weeds *Eclipta prostrata* and *Veronica persica* at 400 μg·mL^−1^, and the growth inhibition rates were 49.7 and 18.4%, 81.7 and 24.6%, and 63.7 and 20.3%, respectively; their growth inhibition rates at 400 μg·mL^−1^ against the monocotyledon weeds *Eclipta crusgalli* and *Apostichopus japonicus* were 12.3 and 11.7%, 21.3 and 19.7%, and 9.8 and 20.2%, respectively (Cao et al., 2019). The mechanism underlying growth inhibition by these itaconic acid derivatives is not clear. A report showed that hexylitaconic acid exerts acetylcholinesterase inhibitory activity (IC_50_ = 1.54 μM) (Kaaniche et al., 2019). Acetylcholine is a well-known neurotransmitter in the cholinergic nervous systems of vertebrates and insects and harbors positive effects on plant growth and development, especially under abiotic stress (Sugiyama and Tezuka, 2011; Qin et al., 2020, 2021; Su et al., 2020). Acetylcholinesterase is in charge of conversion of acetylcholine to choline and acetic acid. Thus, the abovementioned inhibitory effect of hexylitaconic acid on acetylcholinesterase might increase the level of acetylcholine, further maintaining acetylcholine function under drought stress. Maybe itaconic acid exerts such an effect to increase acetylcholine level in the needles of I_D seedlings, further maintaining physiological activity in their needles under drought stress. Anyway, such high accumulation of itaconic acid improves more research on its function in plants under drought stress. Furthermore, the metabolite itaconic acid was in significantly positive correlations to the four proteins, i.e., A0A023W8I6, A0A023W8D4, C3VXI1, and C6ZG55 (the file “I_D.vs.NI_D_neg_I_D.vs.NI_D_cor” in Supplementary Table S16). A0A023W8I6 is dehydrin 1 in *Pinus echinata* (Shortleaf pine), A0A023W8D4 is the protein LP3-3 in *Pinus elliottii*, C3VXI1 and C6ZG55 are large ribosomal subunit protein uL22c and 1-deoxy-D-xylulose-5-phosphate synthase in *P. taeda*, respectively (See Foot note text 2). Therefore, high accumulation of itaconic acid in the needles of I_D seedlings suggests that the metabolite might upregulate the expression of water deficient-inducible proteins (dehydrin 1 and LP3-3) and at the same time maintain the ribosomal function (the protein uL2cc) and carbohydrate conversion (1-deoxy-D-xylulose-5-phosphate synthase). In view of high accumulation of itaconic acid in the needles of *P. taeda* seedlings under drought stress, it is necessary to explore its function in plants under drought stress.

### 4.3 *Serendipita indica*-induced drought tolerance by increasing levels of drought-related proteins in *Pinus taeda*

*Serendipita indica* inoculation caused different pattern changes in differential proteins in *P. taeda* seedlings under drought stress, compared to those in NI_D seedlings. Two aspects occurred in the pattern changes. First, in the comparison of NI_D vs. NI_W, there were five proteins (H9V821, A0A385JF21, H9V5W8, K7NJV0, and H9WW19) only occurring in the needles of NI_D seedlings (the file “NI_D.vs.NI_W.diff_prot” in Supplementary Table S10), but in the comparison of I_D vs. NI_D, there were two proteins (H9X056 and H9VDW5) only occurring in the needles of I_D seedlings (the file “I_D.vs.NI_D.diff_prot” in Supplementary Table S10). On the other hand, *S. indica* inoculation under drought stress did not increase FCs of H9V821, A0A385JF21, H9V5W8, K7NJV0, and H9WW19 (the file “NI_D.vs.NI_W.diff_prot” and the file “I_D.vs.NI_D.diff_prot” in Supplementary Table S10) but increased levels of two other proteins (H9X056 and H9VDW5). The results suggest that this inoculation changed adaptation mechanisms of *P. taeda* by upregulating levels of unique proteins under drought stress. Second, *S. indica* inoculation caused upregulation of 47 differential proteins in *P. taeda* seedlings under drought stress (the file “I_D.vs.NI_D.diff_prot” in Table S10), but 32 differential proteins were upregulated in non-inoculated seedlings (the file “NI_D.vs.NI_W.diff_prot” in Supplementary Table S10). Among the 79 differential proteins mentioned above, only two proteins were the same, i.e., H9V5W8 and Q41096. H9V5W8 is an uncharacterized protein, and Q41096 is a water deficit stress-inducible protein (LP3-2) (See Foot note text 3). These results suggest that *S. indica* inoculation caused significant changes in species and number of differential proteins in needles of *P. taeda* seedlings under drought stress. Interestingly, five differential proteins, i.e., H9WT28, H9X056, H9VCM7, H9VDW5, and K7NLQ9, did not occur in the NI_D seedlings but appeared in NI_W seedlings (the file “NI_D.vs.NI_W.diff_prot” in Supplementary Table S10), and two of the five proteins, i.e., H9X056 and H9VDW5, again occurred in I_D seedlings (the file “I_D.vs.NI_D.diff_prot” in Supplementary Table S10). The strange situation seems to indicate that better water situation occurred in I_D seedlings. H9X056 is an aspartate transaminase (the file “NI_D.vs.NI_W.diff_prot” and the file “I_D.vs.NI_D.diff_prot” in Supplementary Table S10). H9X056 catalyzes the reaction: L-aspartate +2-oxoglutarate = oxaloacetate + L-glutamate. In the reaction, the product L-glutamate is a very important and multifunctional amino acid (Forde and Lea, 2007; Liao et al., 2022). As an important neurotransmitter in plants, L-glutamate level increased in plants under drought stress (Wu et al., 2023). Thus, the increased level of H9X056 caused by *S. indica* inoculation under drought stress might improve L-glutamate biosynthesis to increase glutamate function. However, L-glutamate was not detected in the needles of I_D seedlings in positive or negative ionization mode (Supplementary Table S3). The result seems to indicate that L-glutamate was rapidly used up once its biosynthesis. H9VDW5 is a stress-response A/B barrel domain-containing protein (the file “NI_D.vs.NI_W.diff_prot” and the file “I_D.vs.NI_D.diff_prot” in Table S10). According to information from NCIB, the protein class occurs in microbes and plants, but little is known about their functions, especially under abiotic stress. In *Arabidopsis thaliana*, four genes encode stress-response A/B barrel domain-containing proteins, i.e., At5g22580, At2g31670, At1g51360, and At3g17210. At3g17210 encodes heat stable protein 1 (HS1)^4^. Such proteins harbor short chains of amino acids; thus, they maybe function as auxiliary proteins to exhibit enzyme protective effects against abiotic stress (Drira et al., 2023). In addition, under negative ionization mode, the metabolites 4-coumaric acid and LPC18:0 were in significantly negative correlations to H9X056 and H9VDW5 (Supplementary Figure S5D and the file “I_D.vs.NI_D_neg_I_D.vs.NI_D_cor” in Supplementary Table S16), suggesting that the enrichment of the two proteins might downregulate their levels. It is easy to understand why LPC18:0 might be downregulated by the two proteins because LPC18:0 is a saturated fatty acid. The increased levels of unsaturated fatty acids are more useful to maintain membrane fluidity of plant cells under drought stress. The significantly negative correlation between 4-coumaric acid and the two proteins suggests reduction of the metabolite in the needles of I_D seedlings. However, some other experiments showed that 4-coumaric acid accumulated in plants under drought stress (Arabsalehi et al., 2023; Oksana et al., 2023). Its reduced level may be related to difference in plant species because Arabsalehi et al. (2023) found decrease in level of quercetin, which often accumulated high levels in plants and played important physiological roles under drought stress (Gray et al., 2003; Singh et al., 2021). Therefore, reduction in the levels of 4-coumaric acid and LPC18:0 is reasonable in *P. taeda* seedlings under drought stress. At the same time, H9X056 and H9VDW5 were in significantly positive correlation with three metabolites, i.e., procyanidin B1, trifolirhizin, and tricin 5-*O-*hexoside (Supplementary Figure S5D). As mentioned, the three metabolites played important roles in plants under drought stress. Therefore, the two proteins showed strong effect on biosynthesis of these metabolites mentioned above.

In the comparison of I_D vs. NI_D, FCs of H9VNE7, A0A385JF23, and H9W5R1 were more than 11.0, 5.0, and 3.0, respectively (the file “I_D.vs.NI_D.diff_prot” in Supplementary Table S10), suggesting that the three proteins played important roles in *P. taeda* seedlings under drought stress. H9VNE7 is an uncharacterized protein in *P. taeda* (See Foot note text 3), and no information can be found about its function in NCBI. A0A385JF23 is ribosomal protein L5 in *P. taeda*. Its enrichment in the needles of I_D seedlings suggests its function in maintaining integrity of ribosome and normal protein biosynthesis under drought stress. H9W5R1is acyl-CoA oxidase C-terminal domain-containing protein in *P. taeda*. Acyl-CoA oxidase acts on CoA derivatives of fatty acids with chain lengths from 8 to 18. It catalyzes the first and rate-determining step of the peroxisomal beta-oxidation of fatty acids and a major producer of hydrogen peroxide (Nakajima et al., 2002; Chung et al., 2020). Its enrichment in the needles of I_D seedlings suggests increased fatty acid metabolisms and H_2_O_2_ production.

As mentioned above, vitamin C significantly increased in the needles of I_D seedlings, compared to that in NI_D seedlings. At the same time, vitamin C was in significantly positive correlations to the four proteins, i.e., A0A023W8D4, H9V6D7, A0A2D1UL07, and H9V5W8 (the file “I_D.vs.NI_D_pos_I_D.vs.NI_D_cor” in Supplementary Table S16). A0A023W8D4 is water stress-inducible protein 3 (LP3-3) in *Pinus elliottii* (Slash pine), and H9V6D7, A0A2D1UL07, and H9V5W8 are dirigent protein, dehydrin 5, and uncharacterized protein in *P. taeda*, respectively (See Foot note text 3). Dirigent protein subfamily has an important function in phenol metabolism (Meng et al., 2023) and modulates cell wall metabolisms during abiotic stress (Paniagua et al., 2017). *ScDir*, a dirigent-like gene in sugarcane, responded to drought and salt stress (Guo et al., 2012). The protein ZmESBL, a dirigent family protein in maize, confers variation of Casparian strip thickness and salt tolerance in maize (Wang et al., 2022). Thus, H9V6D7 should have the same function in *P. taeda* under drought stress. Therefore, these proteins showed synergistic roles with vitamin C.

In addition, some function-known and water stress-related proteins were upregulated in I_D seedlings, such as A0A023W8I6 (dehydrin 1) and A0A2D1UL07 (dehydrin 5), compared to their levels in NI_D seedlings (the file “I_D.vs.NI_D.diff_prot” in Supplementary Table S10). These upregulated proteins increased drought tolerance of the inoculated seedlings. Furthermore, as mentioned above, four water deficit-inducible proteins, i.e., Q41087, Q41095, Q41096, and A0A023W8D4, occurred in nuclei (the file “I_D.vs.NI_D.Subcellular_diff” in Supplementary Table S14). Q41087, Q41096, and Q41095 are LP3-1, LP3-2, and LP3-3 in *P. taeda*, respectively; A0A023W8D4 is LP3-3 in *Pinus elliottii* (See Foot note text 3). LP3 paralogs in *P. taeda* belong to the abscisic acid and water stress-induced protein family and act as transcription factors for genes involved in hexose transport, and their levels were upregulated in *P. taeda* under drought stress (Lecoy et al., 2023). Therefore, our results about the subcellular localization of LP3 paralogs are reasonable. Since they act as transcription factors, their targeted genes are still not clear.

### 4.4 Changes in metabolism pathways caused by *Serendipita indica* inoculation in *Pinus taeda* under drought stress

As mentioned above, the levels of some metabolites were increased in the needles of I_W and I_D seedlings, suggesting the related biosynthesis pathways were strengthened. In the comparison of NI_D vs. NI_W seedlings, there were more differential proteins occurring in ribosome and phenylpropanoid biosynthesis pathway, with higher test reliability (Figure 6B), 9 and 5 differential proteins in the two pathways, respectively (the file “NI_D.vs.NI_W.KEGG_Enrich” in Supplementary Table S12). Nine differential proteins in ribosome pathway included R4LC26, H9VPZ0, H9VBV6, H9V5C5, H9WG89, C3VXI1, H9V4M9, H9WZ62, and R4L654 (the file “NI_D.vs.NI_W.KEGG_Enrich” in Supplementary Table S12). These proteins are structural constituents of ribosomes (See Foot note text 3). Out of them, R4LC26, C3VXI1, and R4L654 are involved in rRNA binding. The enrichment of these proteins under drought stress suggests that protein biosynthesis increases to maintain cell physiological functions. The phenylpropanoid biosynthesis pathway produces a great number of important organic compounds with an aromatic ring and a three-carbon propene tail, and they are synthesized by plants from the amino acids phenylalanine and tyrosine (Zhang and Stephanopoulos, 2016). Its general pathway begins from phenylalanine, which is converted to cinnamic acid by phenylalanine ammonia-lyase, and then many compounds were converted, such as coumarins, benzoic acids, anthocyanins, flavonoids, and stilbenes (Vanholme et al., 2019). In phenylpropanoid biosynthesis pathway, the five proteins, i.e., H9VFU4, H9VCR5, H9XAC0, H9WR60, and H9XB10, were enriched (the file “NI_D.vs.NI_W.KEGG_Enrich” in Supplementary Table S12). H9VFU4, H9XAC0, and H9WR60 are plant heme peroxidases and are involved in response to oxidative stress. Their enrichment is useful for scavenging of reactive oxygen species, and the related reaction products are used for biosynthesis of metabolites. H9VCR5 is a phenylalanine ammonia-lyase and is involved in cinnamic acid biosynthetic process. Cinnamic acid and its derivatives harbor antioxidant and antimicrobial activities (Sova, 2012), and *cis*-cinnamic acid is a natural plant growth-promoting metabolite (Steenackers et al., 2019). Thus, the increased levels of cinnamic acid and its derivatives caused by H9VCR5 enrichment are helpful for *P. taeda* seedlings under drought stress. H9XB10 is a glycoside hydrolase and is involved in carbohydrate metabolic process, i.e., hydrolyzing *O*-glycosyl compounds. Its products should be used in phenylpropanoid biosynthesis. Altogether, all the results suggest great changes in metabolism pathways in *P. taeda* caused by drought stress, compared with suitable water condition.

Similarly, some changes in metabolism pathways occurred were caused by *S. indica* inoculation under drought stress. In the comparison of I_D and NI_D seedlings, the first three KEGG enrichment pathways with lower *p*-values were phenylpropanoid biosynthesis, 2-oxocarboxylic acid metabolism, and cutin, suberine, and wax biosynthesis (Figure 6D and the file “I_D.vs.NI_D.KEGG_Enrich” in Supplementary Table S12). In the phenylpropanoid biosynthesis pathway, four proteins, i.e., H9WGR4, H9VCR5, H9XAC0, and H9WR60, were enriched (the file “I_D.vs.NI_D.KEGG_Enrich” in Supplementary Table S12). Out of them, H9WGR4 was a new face and is a plant heme peroxidase. Enrichment of the heme peroxidase suggests that *S. indica* inoculation increased response of *P. taeda* seedlings to oxidative stress under drought stress. As mentioned above, the three proteins, i.e., H9VCR5, H9XAC0, and H9WR60, were enriched in NI_D seedlings. The three proteins were also enriched in I_D seedlings, suggesting *S. indica* inoculation strengthened phenylpropanoid biosynthesis pathway to increase drought tolerance. In the 2-oxocarboxylic acid metabolism pathway, two proteins, i.e., H9VVX5 and H9X056, were enriched in I_D seedlings. H9VVX5 is an uncharacterized protein, and no information is known about its function. As mentioned above, H9X056 was only enriched in I_D seedlings. Its enrichment strengthened L-glutamate biosynthesis. In the cutin, suberine, and wax biosynthesis pathway, the protein, H9WHC3, was enriched (the file “I_D.vs.NI_D.KEGG_Enrich” in Supplementary Table S12). H9WHC3 is also an uncharacterized protein, and no information is known. Since it is involved in cutin, suberine, and wax biosynthesis, its enrichment is useful for plants to reduce water loss from I_D seedlings.

As mentioned above, it is known that *S. indica* inoculation improved two biosynthesis pathways, i.e., phenylpropanoid biosynthesis, and cutin, suberine, and wax biosynthesis, and strengthened a metabolism pathway, i.e., 2-oxocarboxylic acid metabolism, by upregulating the related proteins.

## 5 Conclusion

Our experimental results based on untargeted metabolome and proteome analysis showed inoculation of the root endophytic fungus *S. indica* with *P. taeda* improved biosynthesis of some flavonoids and organic acids under drought stress. These metabolites showed their respective physiological functions in *P. taeda* under drought stress. At the same time, the inoculation increased enrichment of water deficient-inducible proteins (such as LP3-1, LP3-2, LP3-3, and dehydrins) and those proteins involved in ribosomal structures (such as A0A385JF23). Changes in biosynthesis and metabolism pathways caused by the inoculation mainly included phenylpropanoid biosynthesis, cutin, suberine, and wax biosynthesis, and 2-oxocarboxylic acid metabolism. In addition, there were positive relationships between accumulation of some metabolites and enrichment of proteins in *P. taeda* under drought stress. The results provided a strong base for breeding drought-tolerant cultivars of *P. taeda* under global climate change and future research, especially the functions of highly accumulated metabolites (such as itaconic acid) and unique proteins (such as water deficient-inducible proteins localized in the nucleus).

## Data availability statement

The original contributions presented in the study are publicly available. This data can be found here: https://www.cncb.ac.cn under accession numbers: OMIX005953 for metabolome data and OMIX005954 for proteome data.

## Author contributions

CW: Data curation, Funding acquisition, Investigation, Writing – original draft, Writing – review & editing. YY: Investigation, Methodology, Software, Writing – original draft, Writing – review & editing. YW: Data curation, Investigation, Methodology, Software, Writing – original draft, Writing – review & editing. WZ: Resources, Supervision, Writing – review & editing. HS: Funding acquisition, Project administration, Resources, Software, Writing – review & editing.

## Footnotes

^1^ http://string.embl.de/

^2^ http://string-db.org/

^3^ https://www.uniprot.org/uniprotkb/

^4^ https://www.arabidopsis.org/
