# Supplementary material for: Colonization of root endophytic fungus Serendipita indica improves drought tolerance of Pinus taeda seedlings by regulating metabolome and proteome
Source: Front Microbiol. 2024 Mar 15;15:1294833. doi: 10.3389/fmicb.2024.1294833 (PMC10978793; doi:10.3389/fmicb.2024.1294833)
Supplement: Supplementary file 1 [file Data_Sheet_1.zip › 1294833_Sun/1294833_Sun_Data-Sheet-1.docx]

**Supplementary material 1**

**Quality control, screening and identification of differential metabolites**

In order to understand the effects of *S. indica* colonization on non-targeted metabolome in needles of *P. taeda* seedlings under drought stress, differential metabolites were characterized and identified as many as possible (Table S2). Quality control showed that all the samples coming different treatment ways respectively clustered together in positive and negative ionization mode (Fig. 1), suggesting that the analysis method had good reproducibility overall. Both PLS-DA and PCA were used to screen and identify differential metabolites in different treatments (Fig. 2, Fig. 3, and Fig. 4). All the results showed that all the samples respectively clustered together and separated each other between different treatments. Linear regression analysis on R2 and Q2 values showed that the R2 value was respectively greater than Q2 value and that the intercept of Q2 linear equation on y axis was less than 0 (Fig. 5 and Fig. 6), suggesting that PLS-DA model was not used for overfitting and that information about the identified differential metabolites was reliable.

According to PLS-DA analysis, differential metabolites were obtained under different comparisons. Under positive ionization mode, for the comparison of I_W vs. NI_W, the total number of differential metabolites with significant changes was 116, out of which, 47 and 69 metabolites were down- and up-regulated, respectively; for the comparison of NI_D vs. NI_W, 74 and 45 metabolites were down- and up-regulated, respectively; for the comparison of I_D vs. I_W, 40 and 52 metabolites were down- and up-regulated, respectively; for the comparison of I_D vs. NI_D, 43 and 89 metabolites were down- and up-regulated, respectively (Table 1, Table S3). Similarly, under negative ionization mode, differential metabolites were detected (Table 1, Table S3).


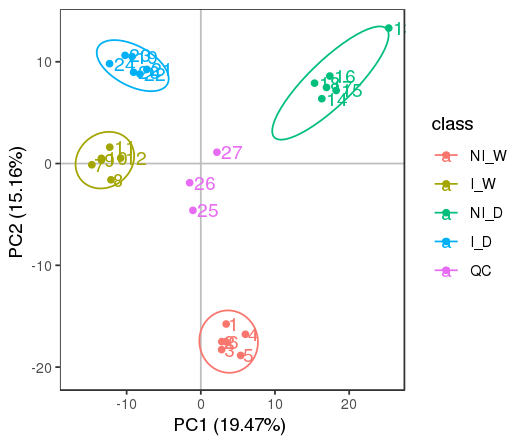

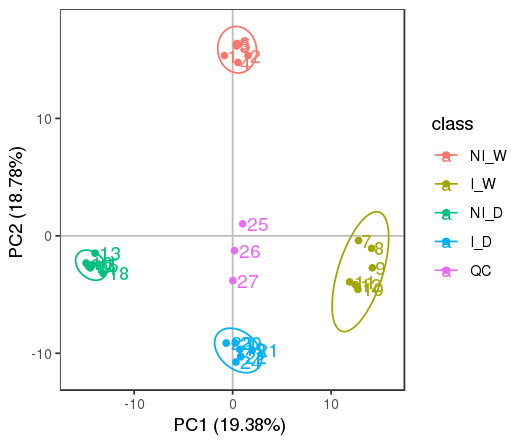


A

B

Fig. 1. PCA score plots based on the UHPLC-Q-TOF/MS data of the needles of *P. taeda* seedlings inoculated (I) or non-inoculated (NI) with *S. indica* under well-watered (W) and drought stress (D) condition, based on positive (A) and negative (B) ionization mode. I_W: inoculated seedlings under well-watered condition; NI_W: non-inoculated seedlings under well-watered condition; I_D: inoculated seedlings under drought stress; NI_D: non-inoculated seedlings under drought stress.


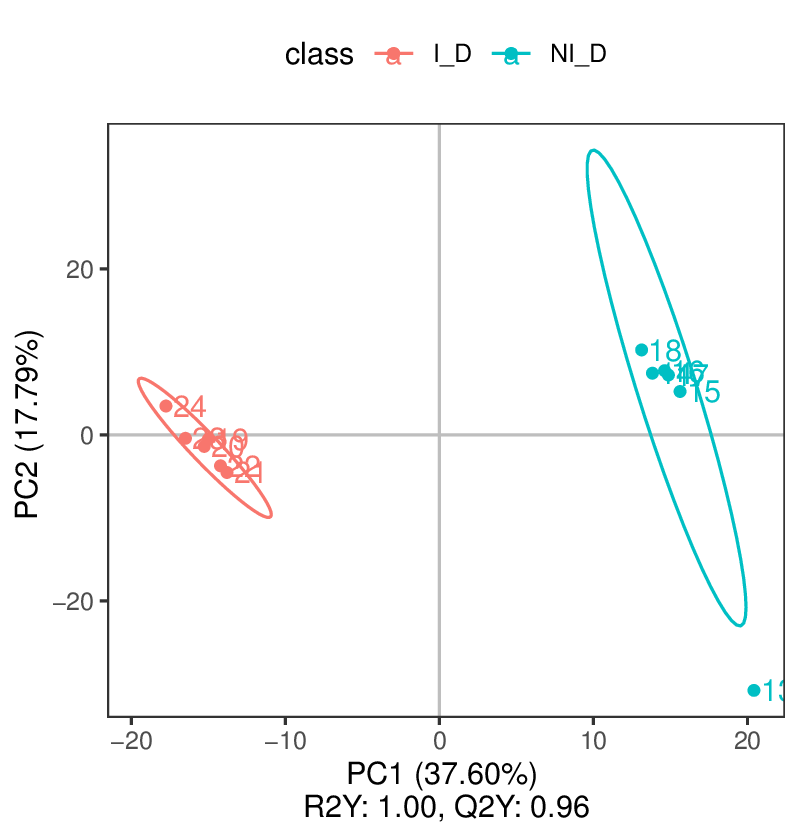

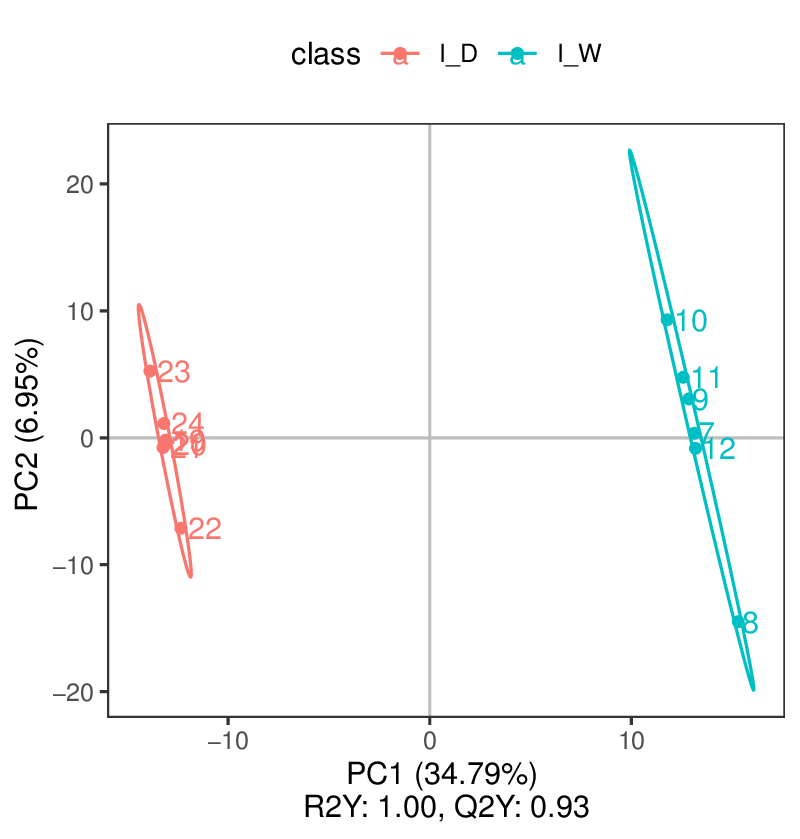

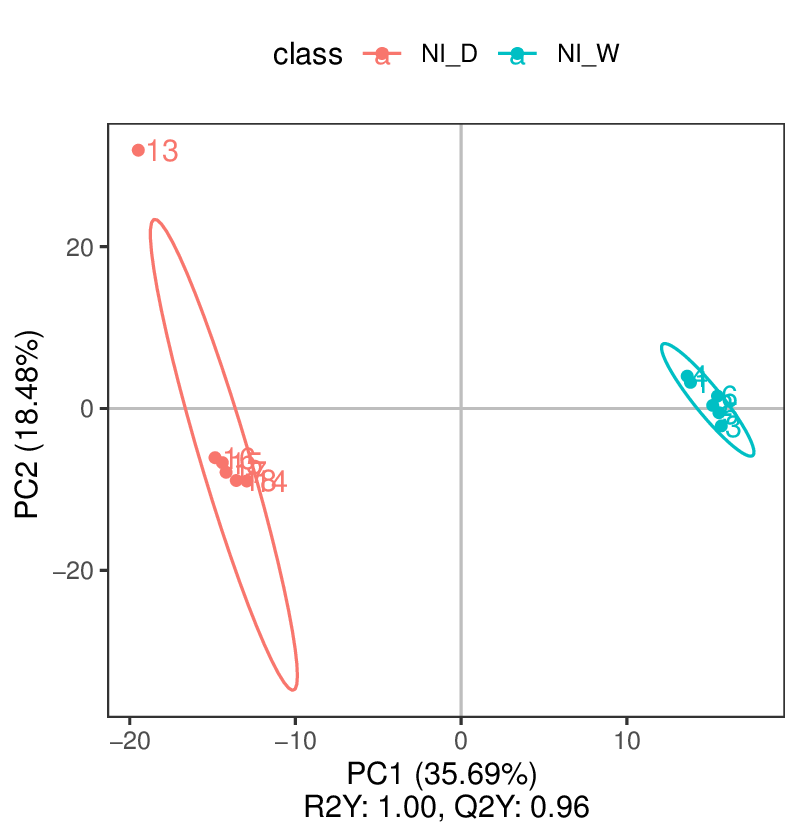

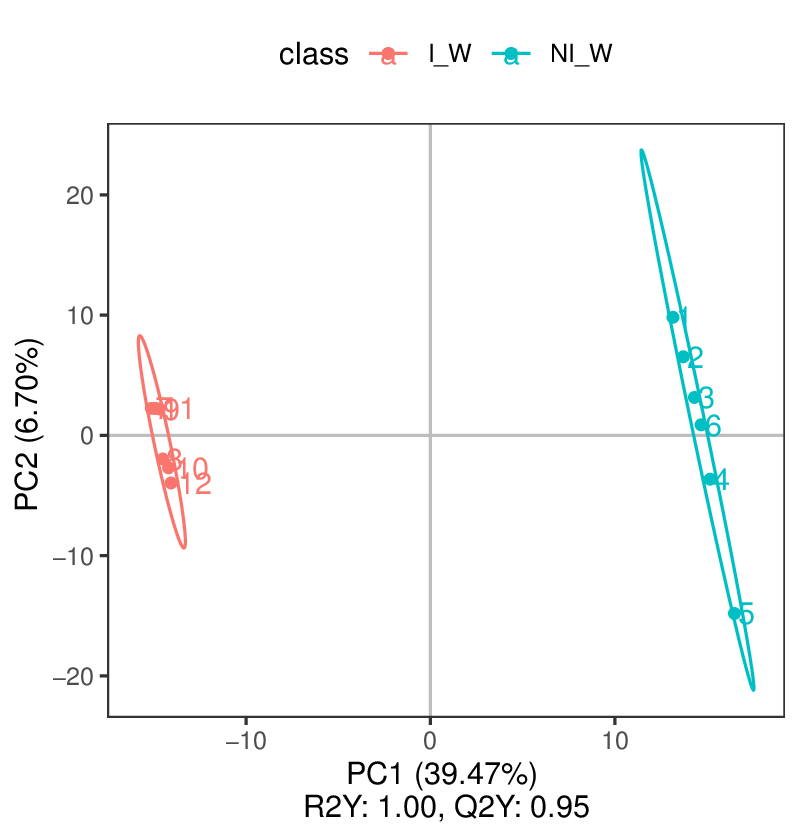


B

D

C

A

Fig. 2. PLS-DA scores of experimental treatments under positive ionization mode. A: I_W (orange) vs. NI_W (blue); B: NI_D (orange) vs. NI_W (blue); C: I_D (orange) vs. I_W (blue); D: I_D (orange) vs. NI_D (blue).I_W: inoculated seedlings under well-watered condition; NI_W: non-inoculated seedlings under well-watered condition; I_D: inoculated seedlings under drought stress; NI_D: non-inoculated seedlings under drought stress.


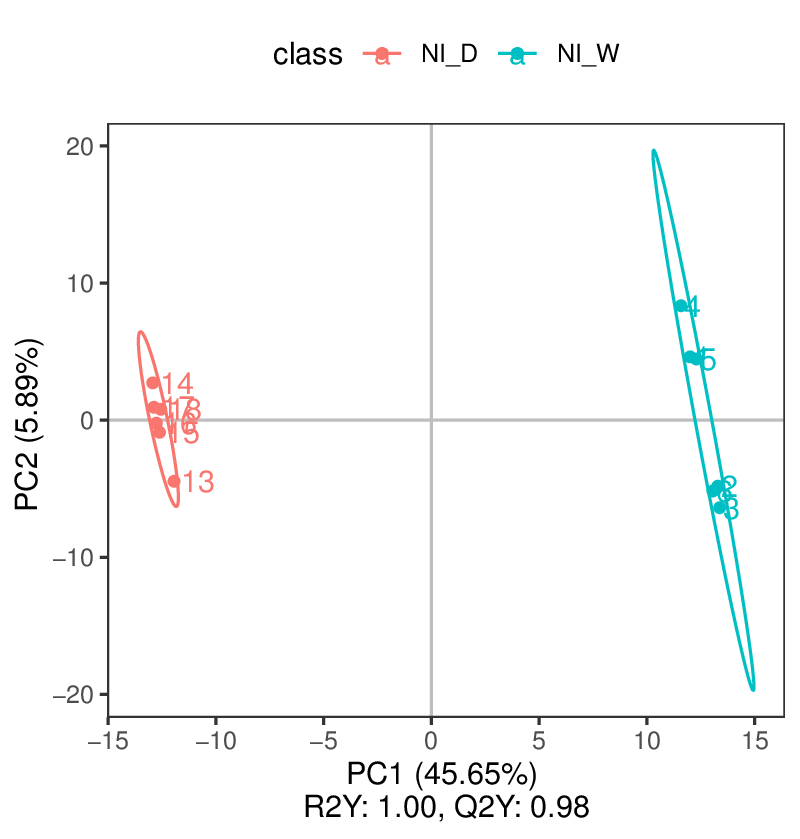

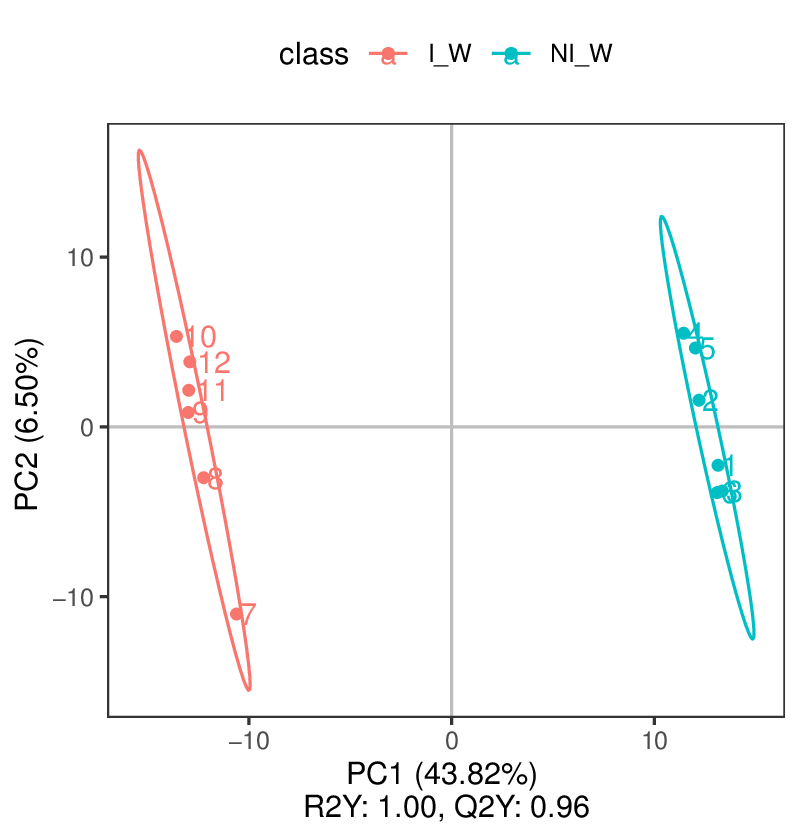


A

B

D


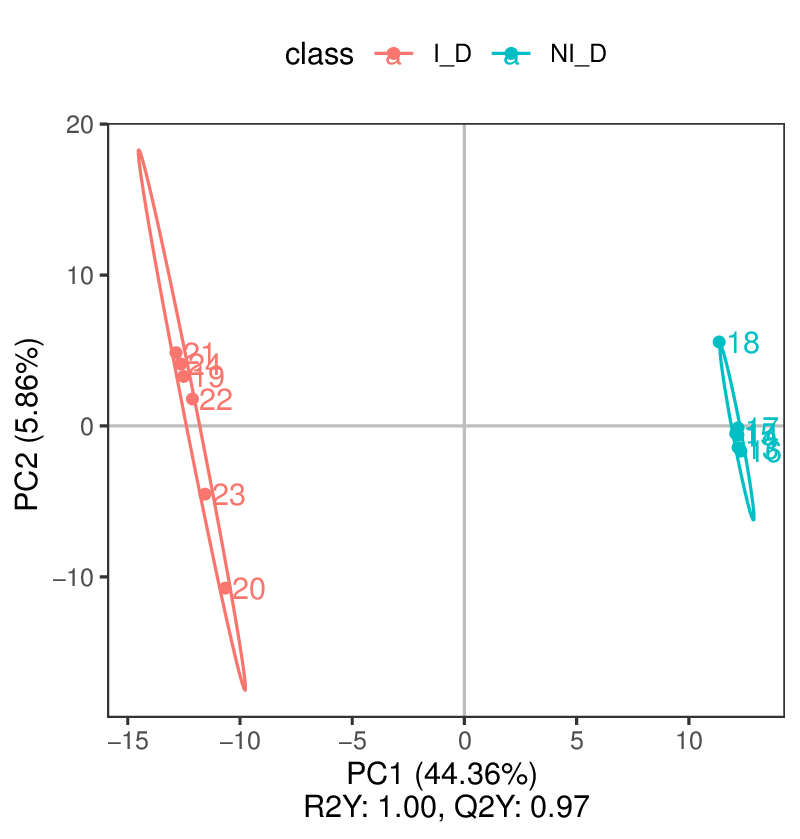

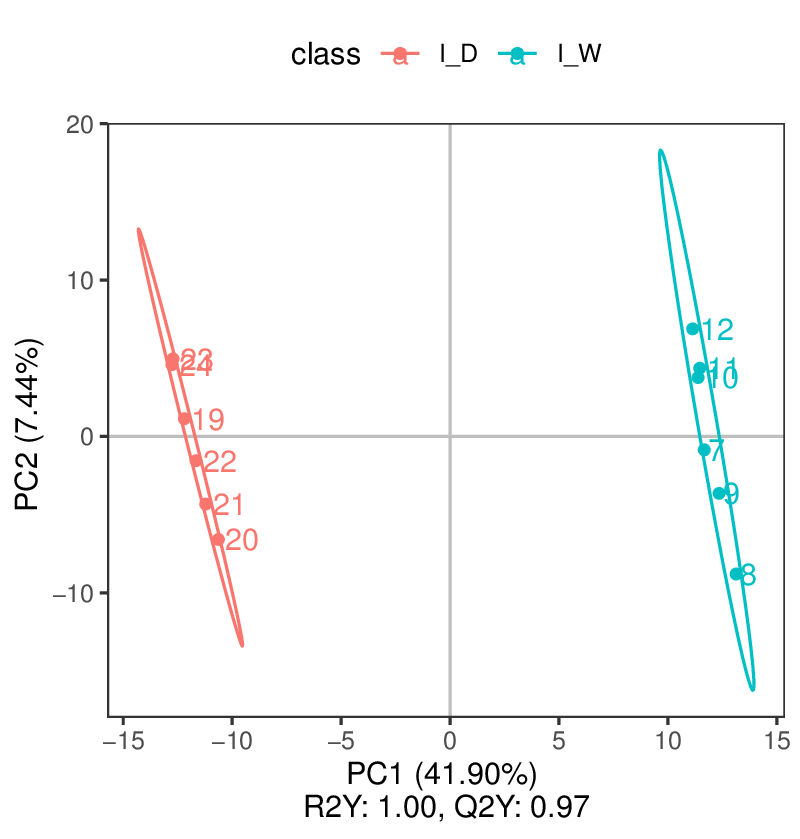


C

Fig. 3. PLS-DA scores of experimental treatments under positive ionization mode. A: I_W (orange) vs. NI_W (blue); B: NI_D (orange) vs. NI_W (blue); C: I_D (orange) vs. I_W (blue); D: I_D (orange) vs. NI_D (blue).I_W: inoculated seedlings under well-watered condition; NI_W: non-inoculated seedlings under well-watered condition; I_D: inoculated seedlings under drought stress; NI_D: non-inoculated seedlings under drought stress.


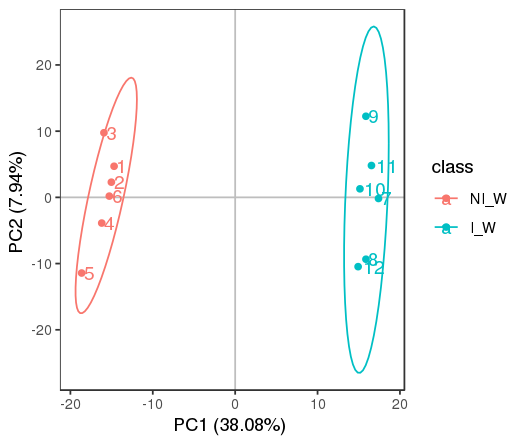

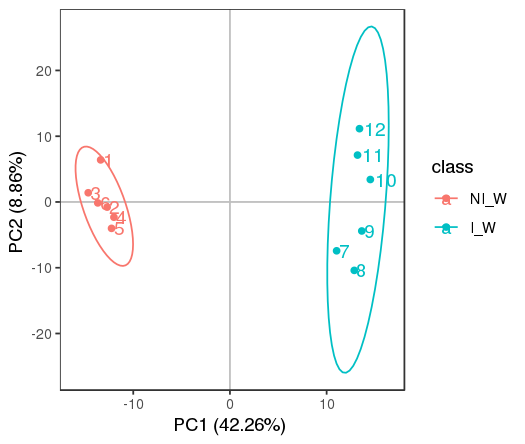


B

A

D


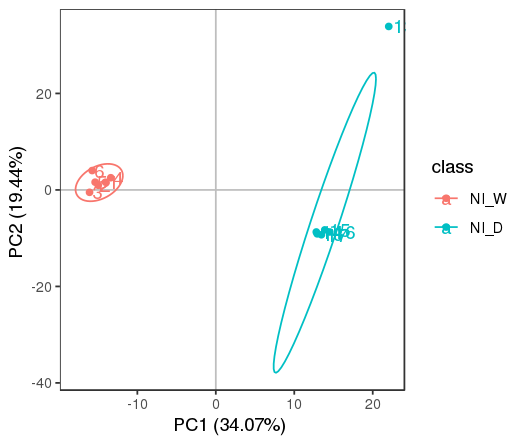

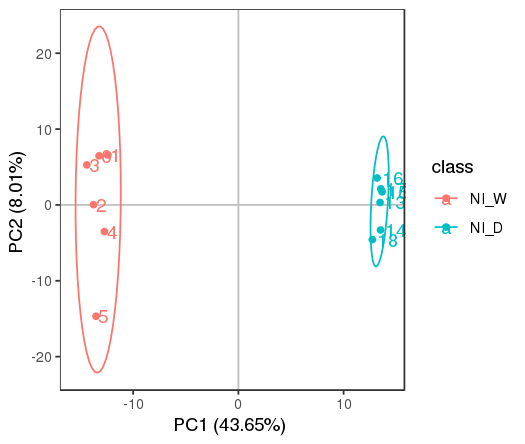


C


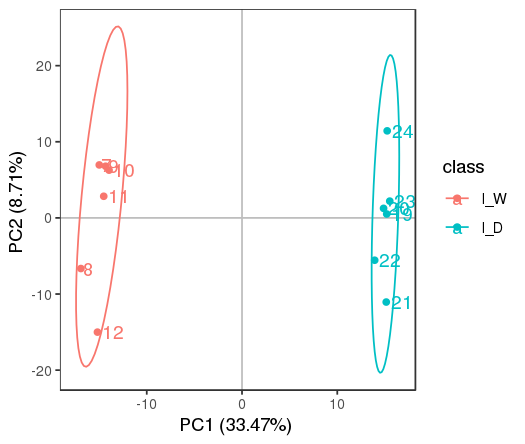

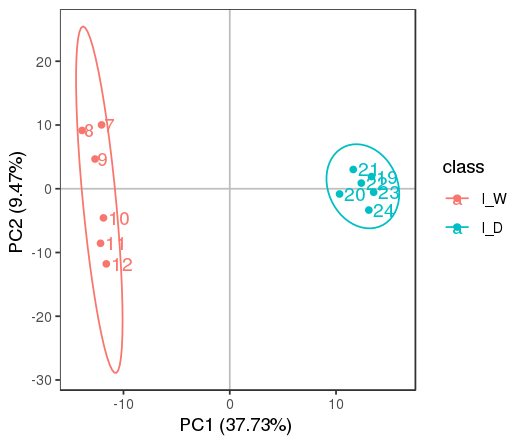


E

H

F


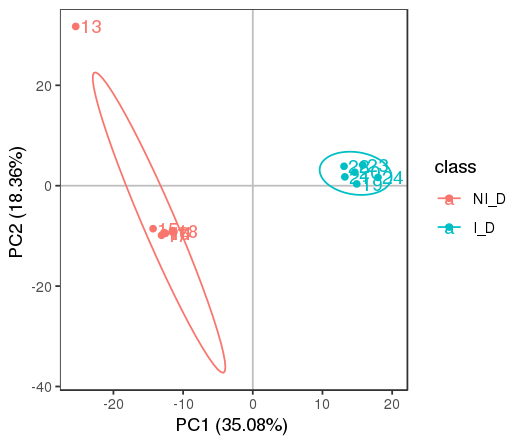

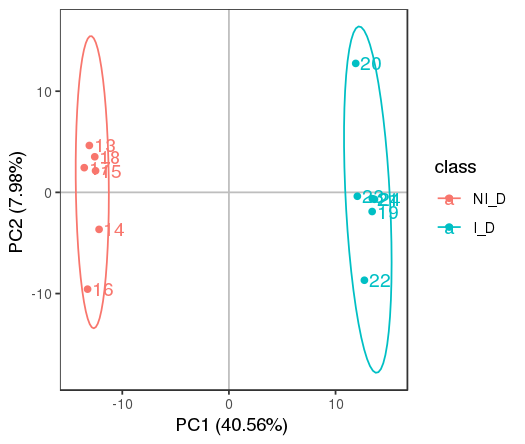


G

Fig. 4. PCA analysis of differential metabolites in samples from different treatments under positive (A, C, E, G) and negative (B, D, F, G) ionization mode. A, B: I_W vs. NI_W; C, D: NI_D vs. NI_W; E, F: I_D vs. I_W; G, H: I_D vs. NI_D. I_W: inoculated seedlings under well-watered condition; NI_W: non-inoculated seedlings under well-watered condition; I_D: inoculated seedlings under drought stress; NI_D: non-inoculated seedlings under drought stress.


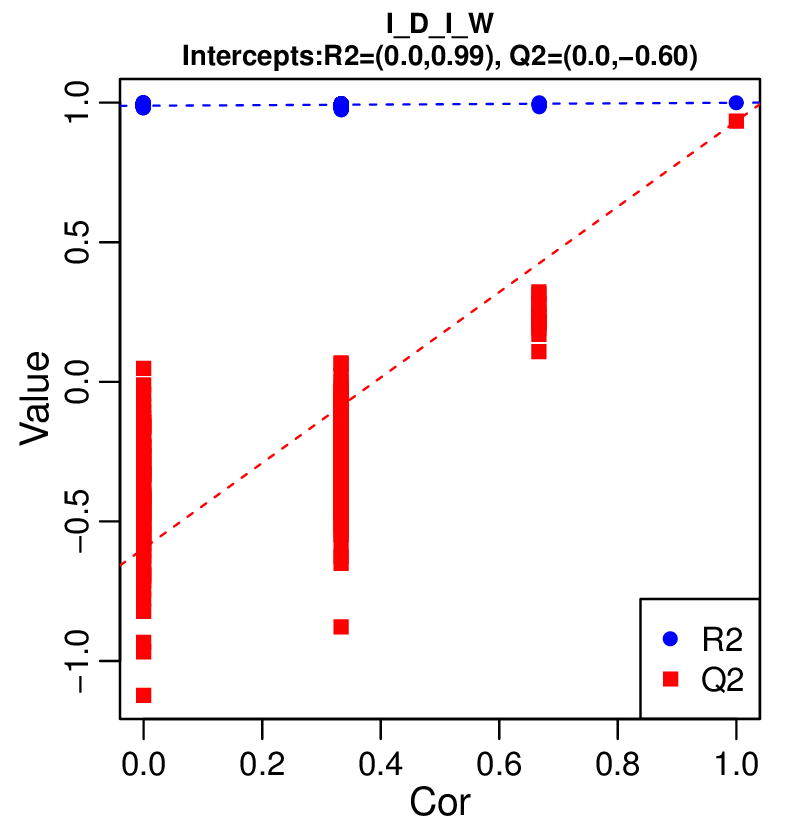

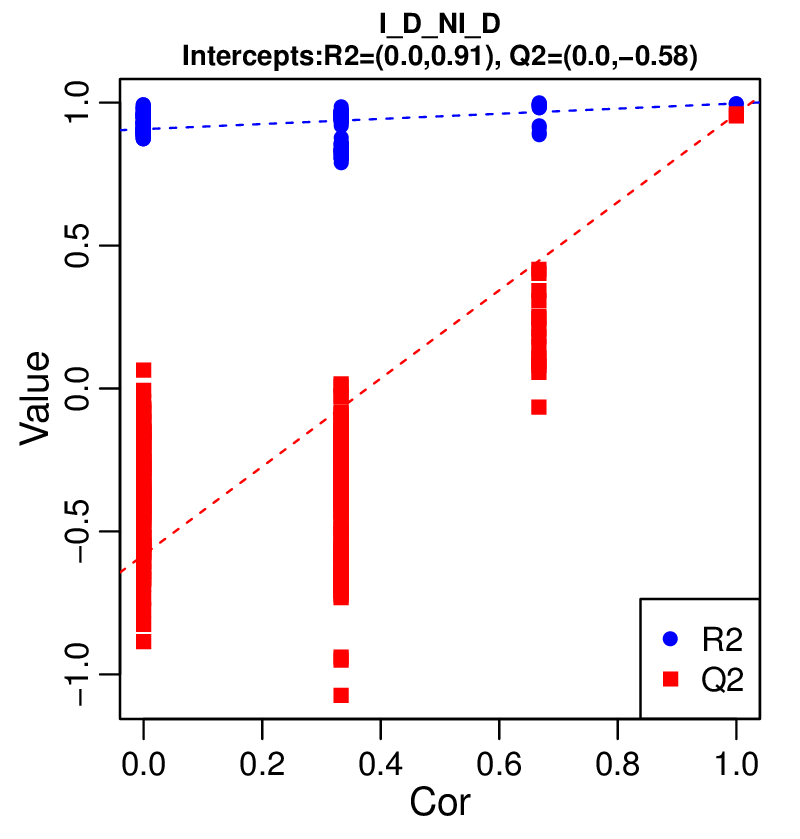

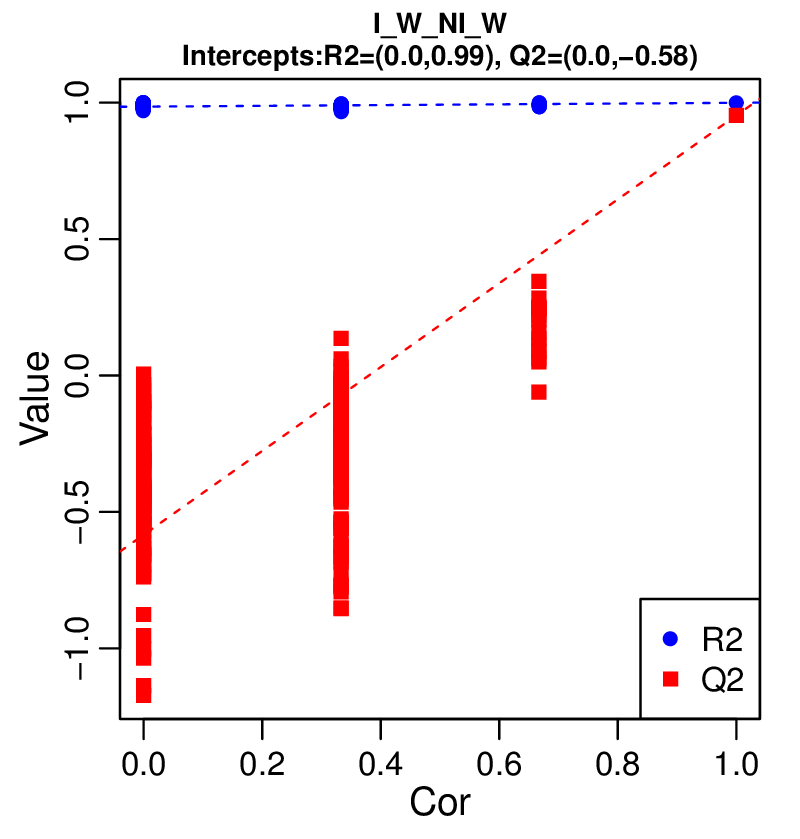

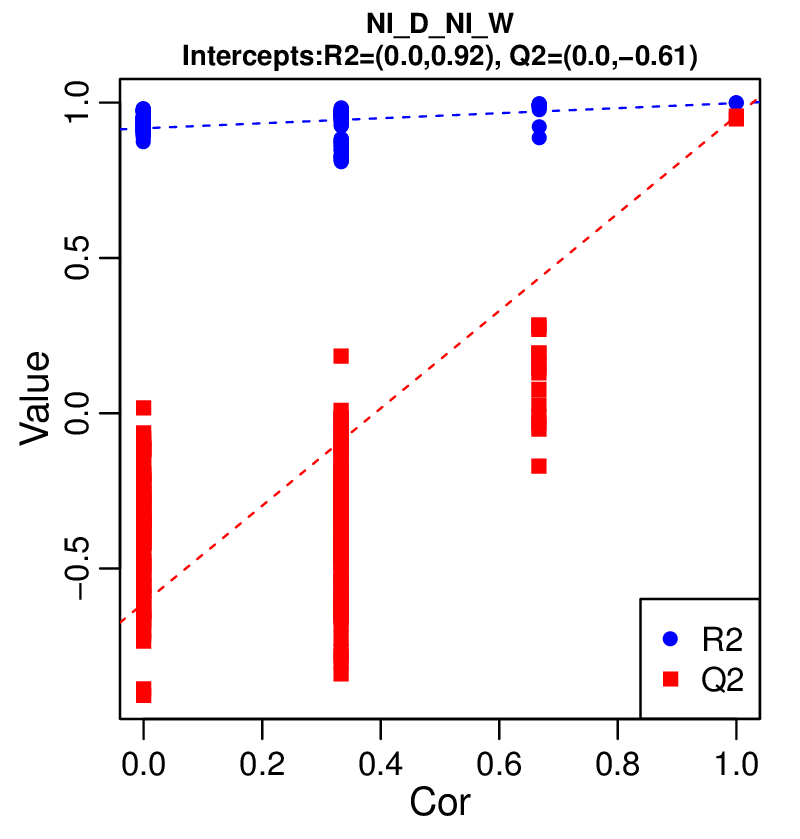


B

A

D

C

Fig. 5. PLS-DA validation test under positive ionization mode. A: I_W vs. NI_W; B: NI_D vs. NI_W; C: I_D vs. I_W; D: I_D vs. NI_D. In each diagram, *x* axis stands for correlation between random component Y and original component Y, and *y* axis stands for scores of R2 and Q2. I_W: inoculated seedlings under well-watered condition; NI_W: non-inoculated seedlings under well-watered condition; I_D: inoculated seedlings under drought stress; NI_D: non-inoculated seedlings under drought stress.

A

D

C

B


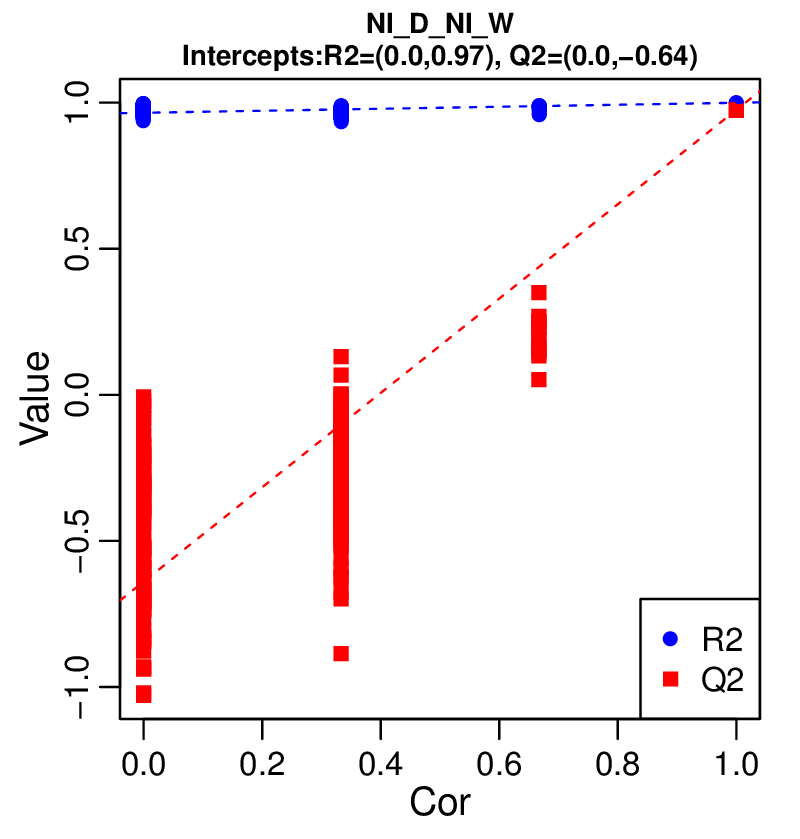

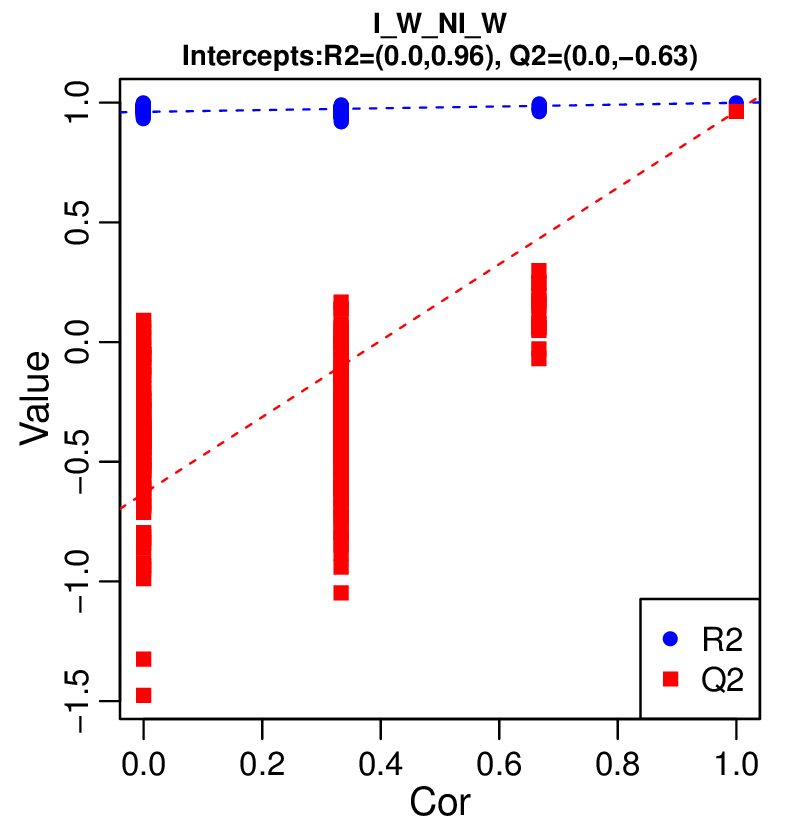


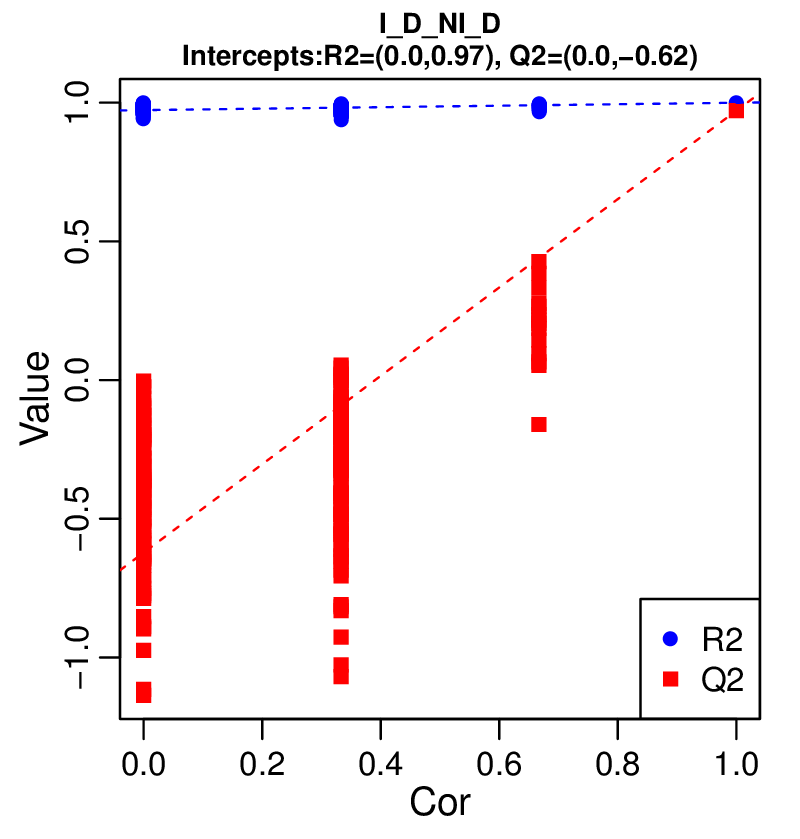

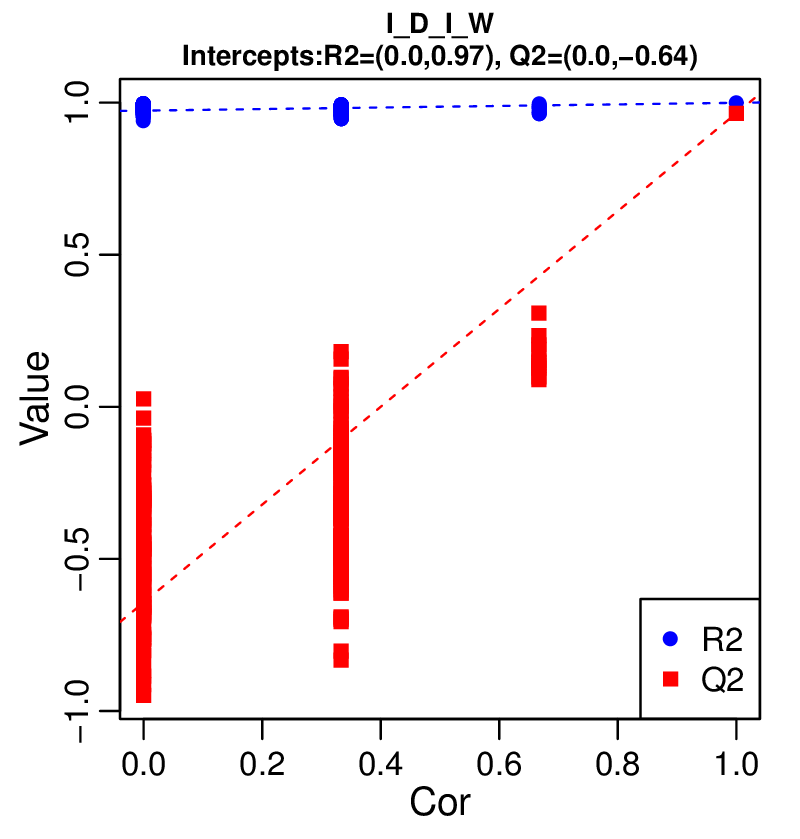


Fig. 6. PLS-DA validation test under negative ionization mode. A: I_W vs. NI_W; B: NI_D vs. NI_W; C: I_D vs. I_W; D: I_D vs. NI_D. In each diagram, *x* axis stands for correlation between random component Y and original component Y, and *y* axis stands for scores of R2 and Q2. I_W: inoculated seedlings under well-watered condition; NI_W: non-inoculated seedlings under well-watered condition; I_D: inoculated seedlings under drought stress; NI_D: non-inoculated seedlings under drought stress.

Table 1. Summary of selective results of differential metabolites.

| Compared samples^1^ | Num. of total ident.^2^ | Num. of total Sig.^3^ | Num. of Sig. down^4^ | Num. of Sig. up^5^ |
| --- | --- | --- | --- | --- |
| I_W. vs. NI_W_pos | 733 | 116 | 47 | 69 |
| NI_D. vs. NI_W_pos | 733 | 119 | 74 | 45 |
| I_D. vs. I_W_pos | 733 | 92 | 40 | 52 |
| I_D. vs. NI_D_pos | 733 | 132 | 43 | 89 |
| I_W. vs. NI_W_neg | 446 | 64 | 23 | 41 |
| NI_D. vs. NI_W_neg | 446 | 67 | 27 | 40 |
| I_D. vs. I_W_neg | 446 | 60 | 26 | 34 |
| I_D. vs. NI_D_neg | 446 | 61 | 24 | 37 |

Note: (1) I_W: inoculated seedlings under well-watered condition; NI_W: non-inoculated seedlings under well-watered condition; I_D: inoculated seedlings under drought stress; I_W: inoculated seedlings under well-watered condition; NI_D: non-inoculated seedlings under drought stress. (2) Total number of identified metabolites; (3) Total number of metabolites with significant differences; (4) Total number of metabolites significantly up-regulated; (5) Total number of metabolites significantly down-regulated. (6) Orange: under positive mode; yellow: under negative mode.
