# Supplementary material for: Colonization of root endophytic fungus Serendipita indica improves drought tolerance of Pinus taeda seedlings by regulating metabolome and proteome
Source: Front Microbiol. 2024 Mar 15;15:1294833. doi: 10.3389/fmicb.2024.1294833 (PMC10978793; doi:10.3389/fmicb.2024.1294833)
Supplement: Supplementary file 1 [file Data_Sheet_1.zip › 1294833_Sun/1294833_Sun_Data-Sheet-2.docx]

**Supplementary material 2**

**Correlation analysis of differential metabolites**

Comparing the differential metabolites in needles of I_W and NI_W seedlings under positive ionization mode, it was found that sedanolide was in positive relation to N3,N4-dimethyle-L-arginine, tangeretin, Vitamin B1, WMH, and 4-guanidinobutanoic acid; that sophoridine was in positive relation to sclareolide, beta-caryophyllene, lysopa 18:0, dihydrocapsaicin, 1-methyladenine; and that 1-methyladenine was in positive relation to Lysopa 18:0, dihydrocapsaicin, vitamin B1 (Fig. 1A, the file “I_W.vs.NI_W_pos_corr” in Table S5). Under negative ionization mode, gibberellic acid was in positive relation to L-ornithine, columbianadin, salicylic acid O-glucoside, and gluconic acid; 2-deoxyglucose-6-phosphate was in negative relation to these differential metabolites. Similarly, wedelolactone, the aflavin, and tracheloside were in negative relation to these metabolites (Fig. 2A, the file “I_W.vs.NI_W_neg_corr” in Table S5). Comparing the differential metabolites in the needles of NI_Dand NI_W seedlings (positive ionizatin mode), it was found that alpha-ketoglutaric acid, rotenone, (-)-alpha-pinene were in positive relation to xanthurenic acid O-hexoside, procyanidin B2,and 4-hydroxybenzoic acid, but was in negative relation to stearoyl ethanolamide, MAG (18:1), and patchouli alcohol (Fig. 1B, the file “NI_D.vs.NI_W_pos_corr” in Table S5). Crocetin was in positive relation to 14-methylhexadecanoic acid, N-lauroylsarcosine, methyl syrinagte, geranylacetone, corymboside, clareolide (Fig. 1B, the file “NI_D.vs.NI_W_pos_corr” in Table S5). Under negative ionization mode, 4-coumaric acid was in positive relation to prostaglandin A3 and prostaglandin H2; Prostaglandin K2 was in postive relation to Prostaglandin A3, Prostaglandin H2, 4-coumaric acid, but in negative relation to (±) 11-HETE and (+) dihydrojasmonic acid (Fig. 2B, the file “NI_D.vs.NI_W_neg_corr” in Table S5). Comparing the differential metabolites in the needles of I_D and I_W seedlings (positive ionization mode), it was found that stachydrine was in positive relation to 14-methylhexadecanoic acid, gardenin B, polypodine B, N-lauroylsarcosine, but in negative relation to sedanolide, arachidonic acid, targinine, and N3,N4-dimethyl_l-arginine (Fig. 1C, the file “I_D.vs.I_W_pos_corr” in Table S5). Under negative ionization mode, trifolirhizin was in positive relation to quercetin-3-O-beta-glucopyranosyl-6´-acetate, eriodictyol O-malonylhexoside, prostaglandin K2, di-C,C-pentosyl-luteolin, hexadecanedioic acid, tricin 5-O-hexoside; and that ingenol was in positive relation to trifolirhizin, tricin 5-O-hexoside, and hexadecanedioic acid (Fig. 2C, the file “I_D.vs.I_W_neg_corr” in Table S5). Gluconic acid was in positive relation to L-ornithine and eleutheroside B, but in negative relation to few metabolites, such as eriodictyol O-malonylhexoside, prostaglandin K2, hexadecanedioic acid, tricin 5-O-hexoside, trifolirhizin, and ptaquiloside (Fig. 2C, the file“I_D.vs.I_W_neg_corr” in Table S5). Rhapontin showed the relation pattern similar to gluconic acid (Fig. 2C). Comparing the differential metabolites in the needles of I_D and NI_D seedlings (positive ionization mode), it was found that the three metabolites, stearoyl ethanolamide, MAG (18:1), patchoulli alcohol, were in negative relation to xanthurenic acid O-hexoside, procyanidin B2, 4-hydroxybenzoic acid; that (-)alpha-pinene, rotenone, alpha-ketoglutaric acid were in positive relation to xanthurenic acid O-hexoside, procyanidin B2, 4-hydroxybenzoic acid, but in negative relation to stearoyl ethanolamide, MAG (18:1), patchoulli alcohol (Fig. 1D, the file “I_D.vs.NI_D_pos_corr” in Table S5). Eriocitrin was the metabolite with the greatest fold change (the file “I_D.vs.NI_D_pos_Diff_order” in Table S3), and it was in positive relation to xanthurenic acid O-hexoside, procyanidin B2, 4-hydroxybenzoic acid, but in negative relation to stearoyl ethanolamide, MAG (18:1), patchoulli alcohol (Fig. 1D, the file “I_D.vs.NI_D_pos_corr” in Table S5). Itaconic acid was the metabolite with the greatest fold change (the file “I_D.vs.NI_D_neg_Diff_order” in Table S3), and it was in positive relation to procyanidin B1, quercetin-3-O-beta-blucopyranosyl-6-acetate, nodakenetintrifolirhizin, tricin 5-O-hexoside, myricitrin, and sattabacin, but in negative relation to periplogenin, 4-coumaric acid, and eleutheroside B (Fig. 2D, the file“I_D.vs.NI_D_neg_corr” in Table S5).


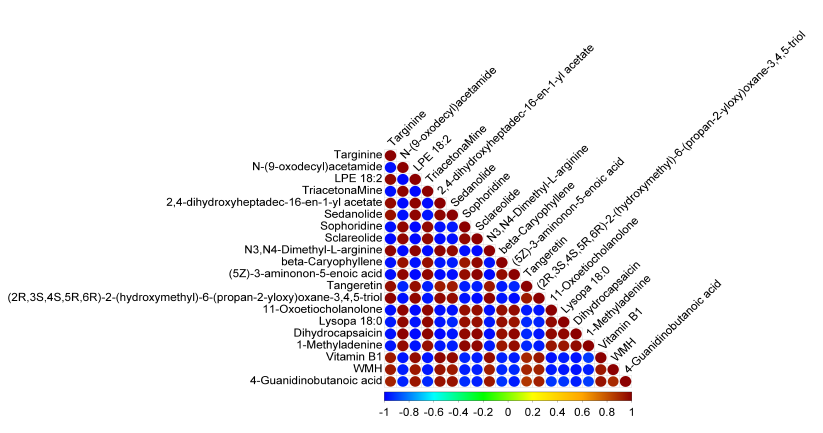


A


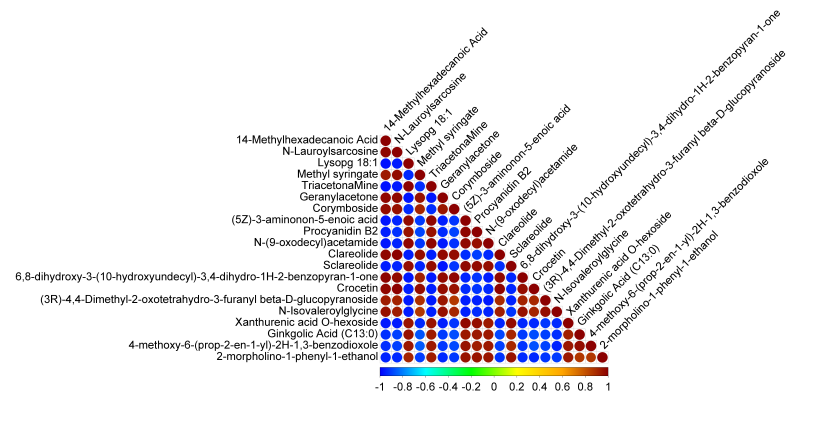


B


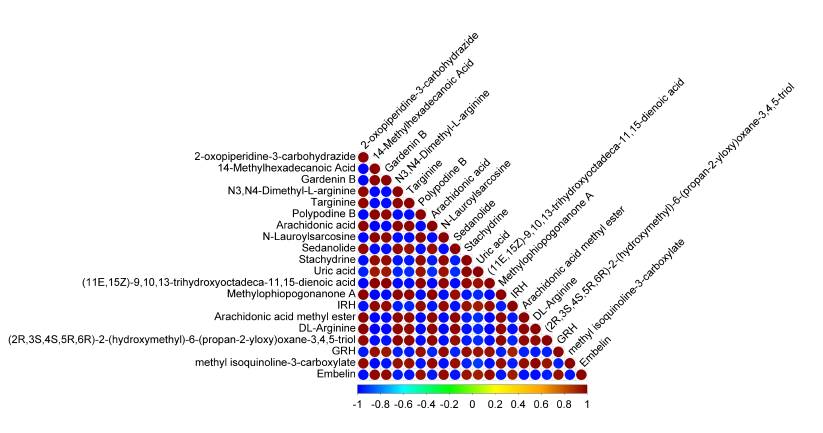


C


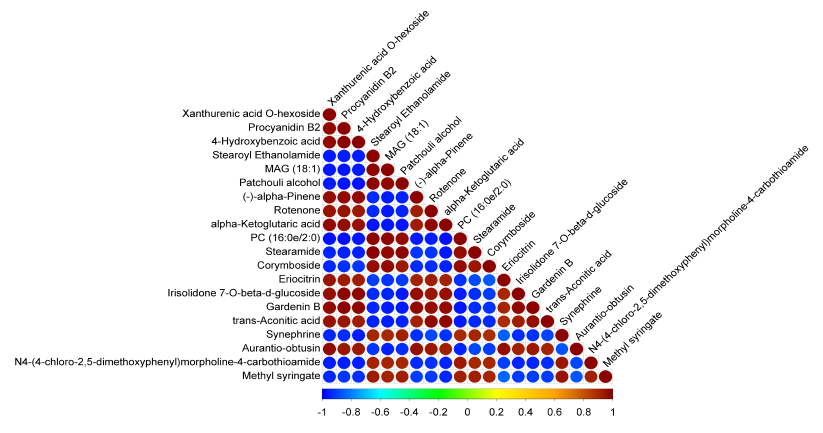


D

Fig. 1. Correlation analysis of metabolites (positive ionization mode) with p values of top 20 (beginning from the minimal *p* value). The highest correlation with correlation coefficient of 1 was represented in red; the lowest correlation with correlation coefficient of -1 was represented in blue. Colorless parts indicated *p* value > 0.05. I_W: inoculated seedlings under well-watered condition; NI_W: non-inoculated seedlings under well-watered condition; I_D: inoculated seedlings under drought stress; NI_D: non-inoculated seedlings under drought stress. A: I_W vs. NI_W; B: NI_D vs. NI_W; C: I_D vs. I_W; D: I_D vs. NI_D.


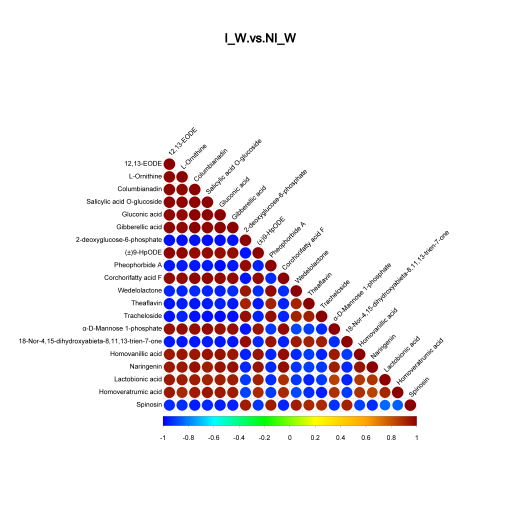


A


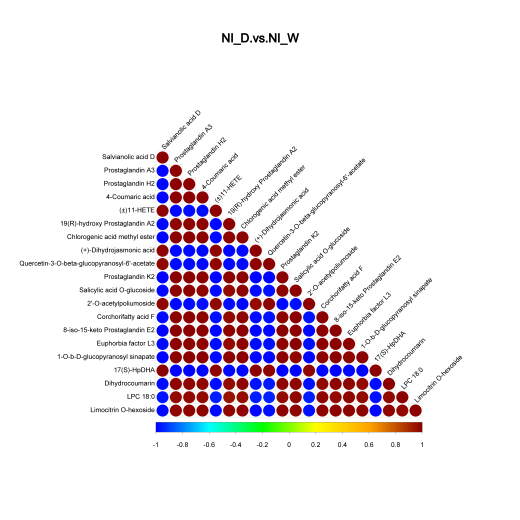


B


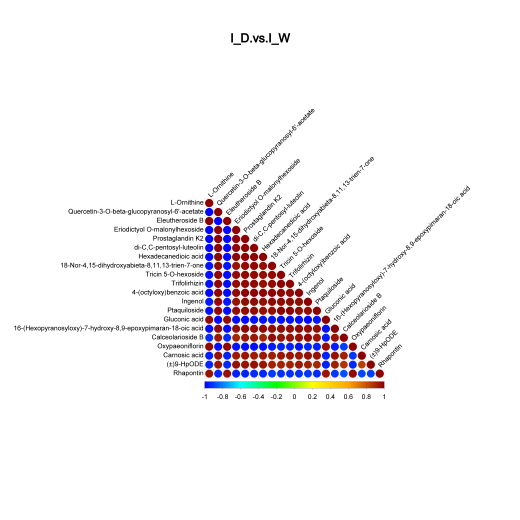


C


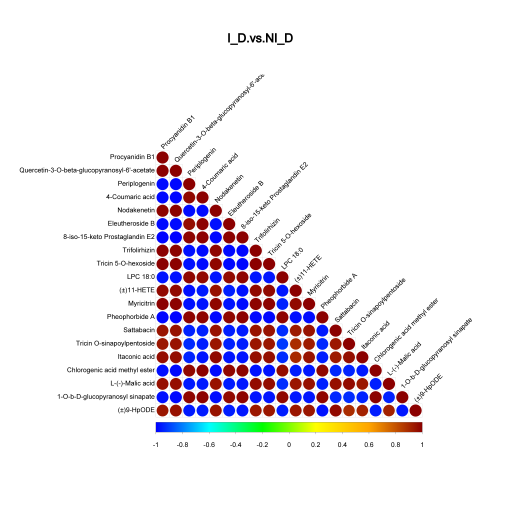


D

Fig. 2. Correlation analysis of metabolites (negative ionization mode) with p values of top 20 (beginning from the minimal *p* value). The highest correlation with correlation coefficient of 1 was represented in red; the lowest correlation with correlation coefficient of -1 was represented in blue. Colorless parts indicated *p* value > 0.05. I_W: inoculated seedlings under well-watered condition; NI_W: non-inoculated seedlings under well-watered condition; I_D: inoculated seedlings under drought stress; NI_D: non-inoculated seedlings under drought stress. A: I_W vs. NI_W; B: NI_D vs. NI_W; C: I_D vs. I_W; D: I_D vs. NI_D.
