# Supplementary material for: Colonization of root endophytic fungus Serendipita indica improves drought tolerance of Pinus taeda seedlings by regulating metabolome and proteome
Source: Front Microbiol. 2024 Mar 15;15:1294833. doi: 10.3389/fmicb.2024.1294833 (PMC10978793; doi:10.3389/fmicb.2024.1294833)
Supplement: Supplementary file 1 [file Data_Sheet_1.zip › 1294833_Sun/1294833_Sun_Data-Sheet-3.docx]

**Supplementary material 3**

**Function annotation and quantitative analysis**

Proteome analysis identified the total number of 793 proteins, out of which 407 proteins were functionally annotated (the file “GO.anno.” in Table S7). GO annotation was shown in Fig. 1 and the file “GO.anno.go2protein” in Table S7. In the section of “Biological Process”, 66 proteins were involved in oxidation-reduction process and 16 proteins in response to stress; in the section of “Cell Component”, 38 proteins were involved in ribosome and 27 in intracellular component; in the section of “Molecular Function”, 40 proteins were involved in structural constituent of ribosome and 32 in ATP binding (Fig. 1).

Quantitative analysis of identified proteins and their sequences were included in Table S8. Principal component analysis showed that samples from different treatments possessed great changes (Fig. 2), suggesting that these different treatments greatly affected protein express. CV cumulative curve showed that all the samples from different treatments possessed good repeatability (Fig. 3). All the results suggest that the data in this proteome analysis are reliable.


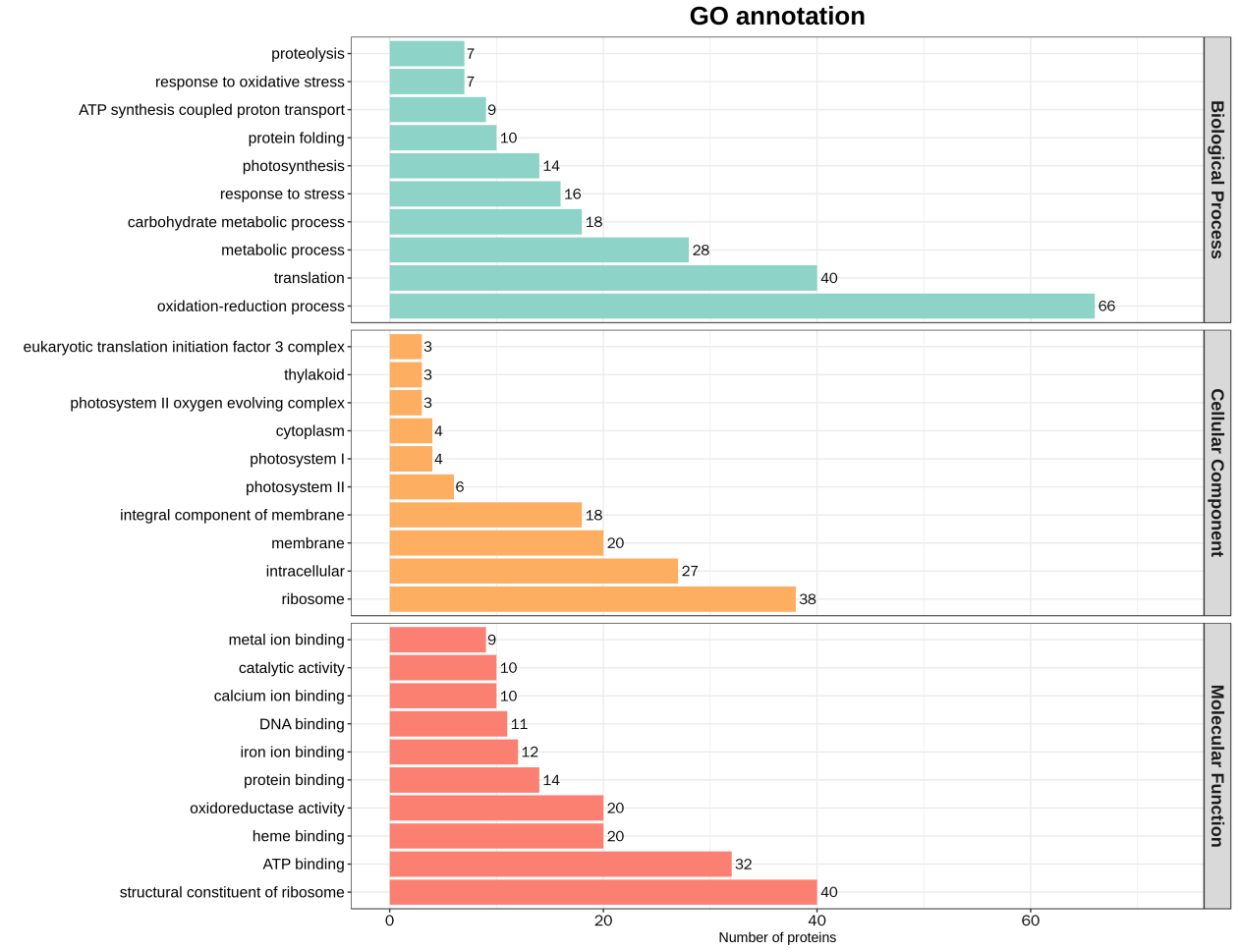


Fig. 1. Diagram of GO annotation. In this diagram, *x* axis stands for the numbers of proteins, and *y* axis stands for GO items.


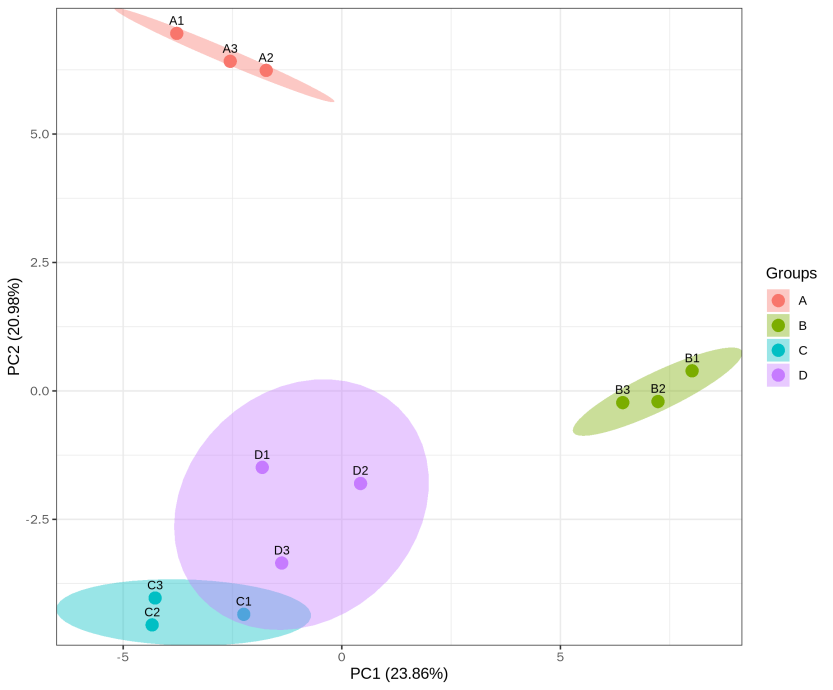


Fig. 2. PCA analysis of proteins in experimental treatments. A: non-inoculated and watered seedlings; B: inoculated wand watered seedlings; C: non-inoculated and drought-treated seedlings; D: inoculated and drought-treated seedlings.


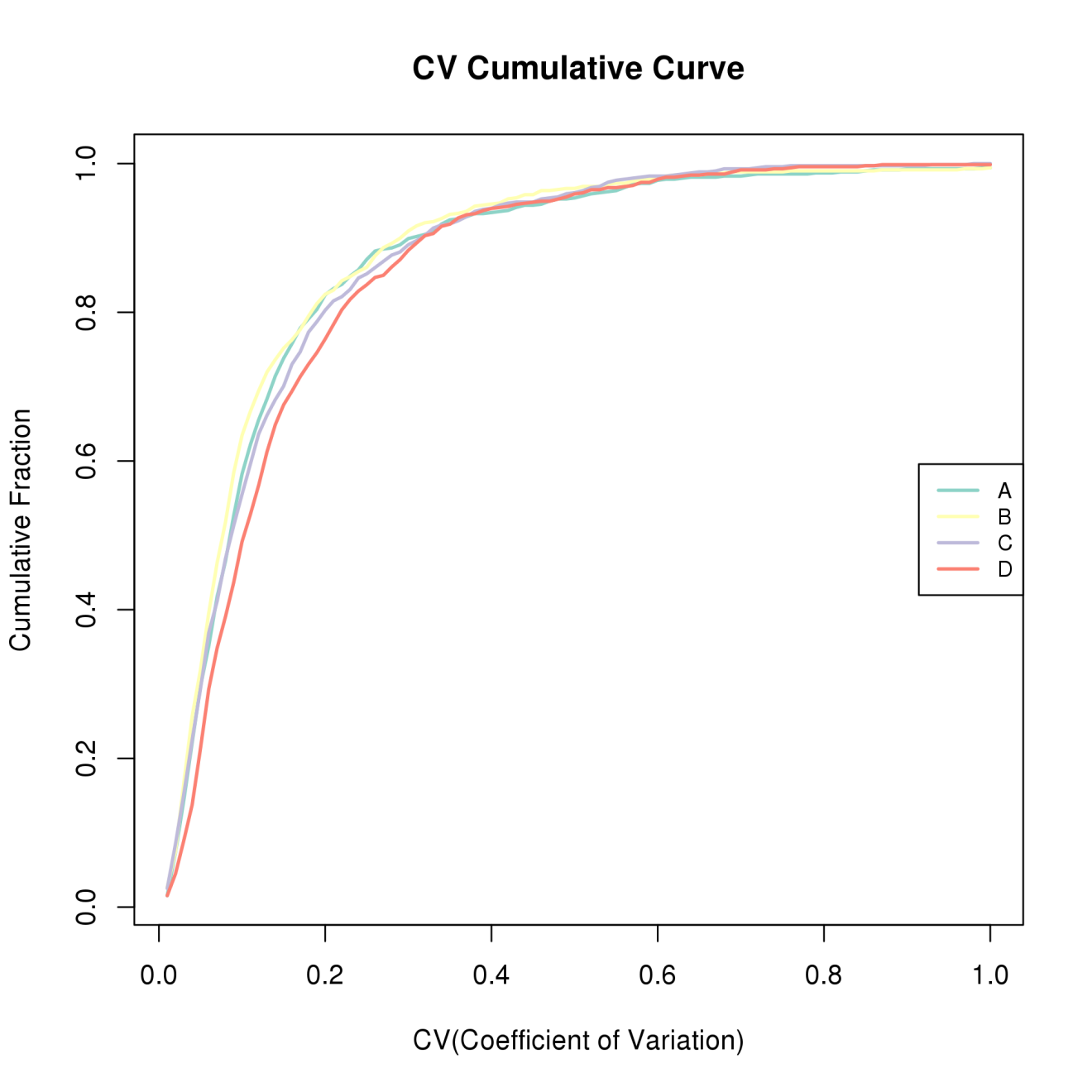


Fig. 3. CV cumulative curves of samples from different treatments. A: I_W vs. NI_W; B: NI_D vs. NI_W; C: I_D vs. I_W; D: I_D vs. NI_D.I_W: inoculated seedlings under well-watered condition; NI_W: non-inoculated seedlings under well-watered condition; I_D: inoculated seedlings under drought stress; NI_D: non-inoculated seedlings under drought stress.

Loblolly pine (Pinus taeda) is native to the southeastern United States (US) and is an important forest tree species in the southern mixed pine-oak forest (Kricher and Morrison, 1998) and the Coastal Plain oak-pine forests (Keyser et al., 2016) in the southern and the southeastern US, respectively. The forest species is the most-planted forest tree species in North America and managed for wood production (. Matallana-Ramirez et al., 2021; McKeand et al., 2021). There are approximately 25 million hectares of planted trees in US in 2011, of which loblolly pine represents 10 million hectares, making it the most planted tree species in the country (Robertson et al., 2011). Planted forests account for 22% of all forested area in the southeastern US (Gonzalez-Benecke et al., 2017), and they play a large role in meeting the nation’s wood and fiber demand. Gonzalez-Benecke et al. (2017) predicted that an increase in productivity can be expected for a large majority of the planted loblolly pine stands in the southeastern US during 21th century. Because of its rapid growth and high oleoresins yield, loblolly pine was also introduced to other countries around the world. Terpenoid oleoresins can be converted into rosin and turpentine, which show wide and strong commercial uses such as adhesives, inks, emulsifiers, solvents, fragrances, and resins (da Silva Rodrigues-Corrêa etal., 2012). According to an estate in 2004, approximately 30,000 tons of pineses were consumed only by the flavor and fragrance industry per year to produce a range of products (Swift, 2004), thus such great requirement improves oleoresins production from *Pinus* species around the world. Since loblolly pine was introduced into China in thirties of last century, the forest tree species became more and more important for plantations along with slash pine (*Pinus elliottii*) in southern China, especially the valleys of the Yangtze River and Zhujiang River. About 0.6 million tons of resin was harvested each year in China, accounting for 60% of the global gum resin yield and about half of the worldwide turpentine trade (McConnell et al., 2021; Yi et al., 2021), most of which was harvested from loblolly pine and slash pine.

Along with other environmental factors, such as soil chemistry, annual average temperature, and incident radiation, water availability is one of the principal physical factors limiting primary productivity of terrestrial plants. Water availability was identified as the most influential single variable for estimating the net primary productivity of terrestrial ecosystems (Churkina et al., 1999). Thus drought stress affects the net primary productivity of terrestrial ecosystems, especially forest ecosystems because of their important ecosystem system service functions. Global climate changes affected oleoresin yield of slash pine (Rodrgues and Fett-Neto, 2009), and also affected oleoresin yield of loblolly pine, because the two forest tree species share the same distribution regions all around the world. In fact, drought stress is the principal cause of seedling mortality in pine forests of the southeastern US (Lorenz et al., 2006). Although annual precipitation is enough for growth and development of loblolly pine in southern China, climate change often caused seasonal drought in some areas in this region. For example, severe drought occurred in autumn, winter, and spring in 2009/2010 and 2011/2012 in southwestern China, especially in Yunnan province (Sun et al., 2014). Such severe drought caused death of forest tree seedlings and saplings in this province. In March-June of 2018, severe drought occurred in Fujian province, located in southeastern China (He F et al., 2022). In summer of 2022, great reduction in precipitation occurred in the valley of Yangtze River, resulting in flux break of few tributary rivers and partial occurrence of riverbed of Yangtze River. According to data collected from 1952-2010, Um et al. (2022) confirmed that the propagation phenomenon of meteorological to hydrological and to agricultural droughts occurred in the valley of Yangtze River. Therefore, under the background of climate change, forest ecosystems in the valley of Yangtze River are under threat from severe droughts (Yin et al., 2021).

Plants use different mechanisms to survive under drought stress, including the four aspects: (1) Soil water deficit avoidance (e.g., root exploration, water conservation, phenology); (2) stress avoidance (e.g., osmotic adjustment, root-soil isolation); (3) damage avoidance (i.e., stress tolerance, e.g., leaf orientation, evaporative cooling, root to shoot ratio); (4) damage tolerance (e.g., night-time recovery, heat shock proteins, dehydrins); (5) unadapted (e.g., death, organ loss, permanent damage) (Gilbert and Medina, 2016). During water deficient, plants synergistically use these mechanisms to survive, especially under severe drought stress. Facing drought stress, loblolly pine forest farm managers and tree physiologists have to find suitable ways to maintain survival, growth, and oleoresin yield of loblolly pine. The suitable ways include few aspects: (1) to clearly explore the genetic characteristics of loblolly pine (Lorenz et al., 2011; Wegrzyn et al., 2014; Perera et al., 2018; Caballero et al., 2021) and functions of genes and metabolites related to drought stress (González-Martínez et al., 2006; Lorenz et al., 2006; Talbot et al., 2017; Wu et al., 2023), further providing the base for genetic modification of loblolly pine; (2) Based genetic characteristics of loblolly pine, to breed new cultivars of loblolly pine with stronger drought tolerance (Zapata-Valenzuela et al., 2013; Matallana-Ramirez et al., 2021); (3) to take advantage of benefits from beneficial microbes, especially ectomycorrhizal fungi and root endophytic fungi under drought stress (Piculell et al., 2019; Wu et al., 2019; Frank and Garcia, 2021).

Symbiosis between plants and microorganisms occurs wide in natural ecosystems, and affects plant terrestrializations (Puginier et al., 2022), evolution (Batstone, 2022; van Galen et al., 2023), and tolerance to abiotic and biotic stress (Zeng et al., 2022), and widens the habitability ranges of plants (Muñoz and Carneiro, 2022). Among all the beneficial microorganisms symbiosing with plants, five families of microorganisms have been greatly paid on attentions, i.e., arbuscular mycorrhizal fungi (Kaur et al., 2022; Chen W et al., 2023; Razak and Gange, 2023), ectomycorrhizal fungi (Karlsen-Ayala et al., 2022; Xiao et al., 2023; Jörgensen et al., 2023), root endophytical fungi (Manzur et al., 2022; Sun et al., 2022; Qin et al., 2023), dark septate fungi (Chen S et al., 2023; Gaber et al., 2023; Wang et al., 2023), and plant growth-promopting rhizobacteria (PGPRs) (Ahmad et al., 2022; Gowtham et al., 2022; Zhao et al., 2023). These beneficial microorganisms show strong effects on their plant hosts under drought stress (Ahmad et al., 2022; Gowthwam et al., 2022; Zhao et al., 2023). In review of such functions, they are often used as components of biofilmed biofertilizers (Das et al., 2017; Kumar et al., 2021; Zahra et al., 2023). Biofilmed biofertilizers have emerged as a new improved inoculant technology to improve efficient nutrition uptake, to strengthen management of pests and pathogenic microorganisms, and to sustain soil fertility (Das et al., 2017). However, because of their life traits, i.e., obligate biotroph (Zuccaro et al., 2014), it is difficult to proliferate arbuscular mycorrhizal fungi on a large scale in short time. Ectomycorrhizal fungi, root endophytic fungi, and PGPRs show their advantage in agricultural application because of their facultative biotroph. The root endophytic fungus *Serendipita indica* (i.e., formerly named as *Piriformospora indica*) can colonize in a wide range of plant hosts, such as *Arabidopsis thaliana* (Opitz et al., 2021), *Juglans regia* (Liu et al., 2021), *Oryza sativa* (Ghorbani et al., 2021), *Platycladus orientalis* (Wu et al., 2019), and tomato (*Solanum lycopersicum*) (De Rocchis et al., 2022), and shows strong effects on their plant hosts, especially improving plant nutrition uptake (Wu et al., 2019) and tolerance to drought stress (Liu et al., 2021; Boorboori and Zhang, 2022). However, it is unclear how *S. indica* regulates responses of plant hosts to drought stress in levels of metabolome and proteome, especially forest tree species. The related mechanisms are not still clear, such as the functions of effector proteins secreted by the fungus. Analysis of metabolome and proteome provides outline of changes in metabolites and proteins in plants under drought stress and the related results also provide a guide for future research, especially the functions of small metabolites and unique proteins that accumulate to high levels in plants under drought stress.

As mentioned above, beneficial microorganisms show strong ability to improve plant tolerance to drought stress, however, related studies involved in loblolly pine are less, especially those involved in drought tolerance mechanisms. The root endophytic fungus *S. indica* can be used to improve plant tolerance to drought stress, as shown above. In the present study, *S. indica* was used to inoculate with loblolly pine seedlings, and untargeted metabolome and proteome of their needles were analyzed. Our aims are (1) to know changes in metabolome and proteome caused by inoculation of *S. indica* under drought stress; (2) to explore the related mechanisms that are involved in increased drought tolerance caused by *S. indica*.
